# Geriatrische Forschung in Deutschland: Herausforderungen und Chancen – Erkenntnisse aus der GERisearch-Umfrage

**DOI:** 10.1007/s00391-025-02528-z

**Published:** 2025-11-26

**Authors:** Bendix Labeit, Maela Caudal, Varvara Moskiou, Stefan Grund, Thea Laurentius, Johannes Trabert, Olaf Krause, Anna Maria Affeldt, Maximilian König

**Affiliations:** 1 Klinik für Neurologie, Heinrich-Heine-Universität Düsseldorf, Düsseldorf, Deutschland; 2 Altersmedizinisches Zentrum Köln, Cellitinnen-Krankenhaus St. Marien, Köln, Deutschland; 3 Klinik für Geriatrie und Altersmedizin, Charité-Universitätsmedizin Berlin, Berlin, Deutschland; 4 Geriatrisches Zentrum, Universitätsklinikum Heidelberg, Agaplesion Bethanien Krankenhaus Heidelberg, Heidelberg, Deutschland; 5 Universitätsklinik für Geriatrie, Carl von Ossietzky Universität Oldenburg, Oldenburg, Deutschland; 6 Medizinisch-Geriatrische Klinik, AGAPLESION Markus Krankenhaus Frankfurt, Frankfurt, Deutschland; 7 Institut für Allgemeinmedizin und Palliativmedizin, Medizinische Hochschule Hannover und Zentrum für Medizin im Alter, DIAKOVERE Henriettenstift Hannover, Hannover, Deutschland; 8 Klinik II für Innere Medizin, Uniklinik Köln, Köln, Deutschland; 9 Klinik und Poliklinik für Innere Medizin D – Geriatrie, Universitätsmedizin Greifswald, Walther-Rathenau-Str. 49, 17475 Greifswald, Deutschland

**Keywords:** Clinician scientist, Geriatrie, Mentoren, Akademische Barrieren, Geschlechterunterschiede, Clinician scientist, Geriatrics, Mentors, Academic barriers, Gender disparity

## Abstract

**Hintergrund:**

Für exzellente Altersforschung und die Weiterentwicklung der Geriatrie braucht es Ärztinnen und Ärzte, die sowohl klinisch als auch wissenschaftlich aktiv sind.

**Ziel:**

Untersuchung der Forschungsaktivität, -interessen und -bedingungen geriatrisch tätiger Ärztinnen und Ärzte in Deutschland, um wissenschaftliches Potenzial, strukturelle Hürden und Förderbedarf zu identifizieren.

**Material und Methoden:**

In einer bundesweiten Online-Querschnittserhebung wurden Daten zur beruflichen Situation, zur wissenschaftlichen Tätigkeit sowie zu Forschungsinteressen und -hindernissen erhoben.

**Ergebnisse:**

Von 273 Teilnehmenden war jeweils ein Drittel wissenschaftlich aktiv, nicht interessiert oder grundsätzlich interessiert, jedoch aktuell nicht aktiv. Der größte Teil der wissenschaftlich Aktiven (47 %) arbeitete an Universitätskliniken mit geriatrischem Lehrstuhl, wo zugleich die höchste Forschungszufriedenheit berichtet wurde.

Als häufigste Hürde wurde die mangelnde Vereinbarkeit von Klinik und Forschung genannt (49 %). An nichtuniversitären Kliniken ohne geriatrischen Lehrstuhl waren viele forschungsinteressiert, aber inaktiv; hier dominierten arbeitsplatzbezogene Hindernisse (55 %).

Männer waren häufiger promoviert, habilitiert und wissenschaftlich aktiv. Frauen stellten zwei Drittel der forschungsinteressierten, aber inaktiven Teilnehmenden und zeigten besonderes Interesse an Polypharmazie (59 %) und Ernährungsmedizin (40 %).

**Diskussion:**

Zur Stärkung der geriatrischen Forschung sind gezielte Fördermaßnahmen nötig – insbesondere für Frauen und für Ärztinnen und Ärzte an außeruniversitären Einrichtungen. Wichtige Ansatzpunkte sind Mentoring, geschützte Forschungszeiten und der Ausbau geriatrischer Lehrstühle. Trotz struktureller Barrieren ist das wissenschaftliche Interesse hoch.

**Zusatzmaterial online:**

Zusätzliche Informationen sind in der Online-Version dieses Artikels (10.1007/s00391-025-02528-z) enthalten.

## Abstract (en)

**Background:**

High-quality contributions to international aging research and the advancement of geriatrics in Germany require physicians who are both clinically and scientifically active.

**Objective:**

Investigation of the research activity, research interests and research conditions of physicians working in geriatrics in Germany, in order to identify the scientific potential, structural barriers and areas in need of support.

**Material and methods:**

In a nationwide online cross-sectional survey, data were collected on the professional situation, and research activity, as well as research interests and obstacles.

**Results:**

Among 273 participants one third were scientifically active, one third not interested and one third fundamentally interested but currently inactive. The largest proportion of scientifically active participants (47%) worked at university hospitals with a chair in geriatrics, where the highest research satisfaction was reported.

The most frequently cited barrier was the limited compatibility of clinical work and research (49%). At nonuniversity hospitals without a chair in geriatrics many participants were interested in research but inactive and workplace-related obstacles predominated (55%).

Men more often held a doctoral degree or habilitation and were more often active in research. Women accounted for two thirds of interested but inactive participants in research and expressed particular interest in polypharmacy (59%) and nutritional medicine (40%).

**Discussion:**

Targeted measures are needed to strengthen geriatric research in Germany, particularly to support women and physicians at nonuniversity institutions. Key strategies include mentoring, protected research time and expansion of chairs in geriatrics. Despite structural barriers, interest in research remains high.

## Einleitung

Der demografische Wandel führt in Deutschland zu einem steigenden Anteil älterer Menschen: Zwischen 1990 und 2018 nahm die Zahl der über 67-Jährigen um 54 % auf 15,9 Mio. zu und wird Prognosen zufolge bis 2039 auf mindestens 20,9 Mio. anwachsen [1, 2]. Diese Entwicklung geht mit einem deutlich erhöhten Bedarf an geriatrischer Versorgung einher [3]. Auch angesichts des besorgniserregenden Anstiegs der Pflegekosten, die sich in der letzten Dekade nahezu verdoppelt haben [4], kommt der Geriatrie als zentrales Instrument zur Prävention von Pflegebedürftigkeit eine besondere Bedeutung zu. Systematische Reviews zeigen, dass ein umfassendes geriatrisches Assessment im Krankenhaus die Wahrscheinlichkeit einer Entlassung in die häusliche Umgebung signifikant erhöht und Pflegebedürftigkeit verringern kann [5]. Allerdings stößt die bestehende medizinisch-geriatrische Infrastruktur bereits heute an ihre Kapazitätsgrenzen [6]. Um den Herausforderungen einer alternden Gesellschaft adäquat zu begegnen, sind sowohl innovative Versorgungskonzepte als auch eine wissenschaftlich fundierte Weiterentwicklung der geriatrischen Versorgungsstrukturen unerlässlich. Wissenschaftlich aktive Ärztinnen und Ärzte spielen zudem eine entscheidende Rolle bei der erfolgreichen Translation neuer grundlagenwissenschaftlicher Erkenntnisse in die klinische Praxis [7].

Trotz ihrer herausgehobenen Bedeutung ist die Geriatrie wissenschaftlich nach wie vor unterrepräsentiert [8] und bislang nur an wenigen medizinischen Fakultäten verankert [9], mit negativen Folgen für ihre dynamische Weiterentwicklung und die Nachwuchsförderung [10]. Die deutsche Geriatrie weist zudem eine strukturelle Besonderheit auf: Viele geriatrische Lehrstühle sind nicht an Universitätskliniken, sondern an externen Kliniken angesiedelt. Dadurch sind universitäre Forschungsstrukturen, Netzwerke und Mentoringangebote in der Geriatrie bislang teils nur eingeschränkt vorhanden, was die Forschungstätigkeit insbesondere jüngerer Geriaterinnen und Geriater erschwert.

Beiträge zur internationalen Alternsforschung – ob klinisch, translational oder versorgungsnah – sowie die Weiterentwicklung des Fachgebiets erfordern hochqualifizierte Geriaterinnen und Geriater, die idealerweise klinisch *und* wissenschaftlich tätig sind, also „geriatrische Clinician Scientists“ [11]. Bislang fehlten gesicherte Erkenntnisse zum Status quo, also zu Qualifikationen, Interessen und Barrieren wissenschaftlicher Arbeit in der deutschen Geriatrie. Ziel dieser Studie war es daher, die Perspektiven und Herausforderungen geriatrisch tätiger Ärztinnen und Ärzte zu erfassen, um das wissenschaftliche Potenzial des Fachs sichtbar zu machen und Strategien zur Stärkung von Forschung und Versorgung abzuleiten.

## Methoden

Zur Erfassung von Daten zu Demografie, beruflicher Situation, Karrierezielen sowie zu wissenschaftlichen Rahmenbedingungen, Interessen und Herausforderungen wurde eine standardisierte Online-Umfrage über ein webbasiertes Erfassungstool eingesetzt. Der Fragenkatalog wurde in einer Pilotphase mit 56 Mitgliedern der Nachwuchsgruppe „Junge Geriatrie“ der *Deutschen Gesellschaft für Geriatrie (DGG)* auf Verständlichkeit und Relevanz getestet und anschließend final angepasst. Der finale Fragebogen enthielt Multiple-Choice-Fragen und Rating- bzw. Likert-Skalen, zudem wurden kombinierte Frageformate mit Multiple-Choice-Optionen und Freitextfeldern verwendet. Eine vollständige Fassung des Fragebogens ist im Online-Zusatzmaterial zugänglich.

Eingeladen zur Teilnahme waren alle im Erhebungszeitraum (Oktober 2024 bis Januar 2025) in geriatrischen Einrichtungen in Deutschland tätigen Ärztinnen und Ärzte. Um eine möglichst repräsentative Stichprobe zu erreichen, wurde angestrebt, 250 Teilnehmende aus geriatrischen Kliniken im gesamten Bundesgebiet zu gewinnen, was rund 10 % der 2023 im stationären Bereich tätigen Geriaterinnen und Geriater (*n* = 2298) entspricht [12].

Die Umfrage wurde über verschiedene formelle und informelle Kommunikationskanäle verbreitet, darunter die Signalgruppe der *Jungen Geriatrie*, der Newsletter der DGG, Pausenfolien während des DGG-Kongresses 2024, das Wissenschaftsforum der DGG sowie die *Zeitschrift für Gerontologie und Geriatrie*. Aufgrund der Rekrutierung über DGG-nahe Kanäle war ein Selektionsbias von vornherein zu erwarten, da Geriaterinnen und Geriater ohne Anbindung an die DGG (z. B. in kleineren Kliniken, Rehakliniken oder Praxen) schwerer zu erreichen waren und in der Stichprobe daher möglicherweise unterrepräsentiert sind (Selektionsbias).

### Statistische Auswertung

Gruppenvergleiche erfolgten mit Chi-Quadrat-Tests. Bei multiplen Tests innerhalb einer Kategorie wurde das Signifikanzniveau nach Bonferroni angepasst (z. B. auf 0,002 bei 25 Tests). Eine kategorieübergreifende Korrektur erfolgte nicht. Alle Analysen wurden mit IBM SPSS Statistics (Version 30.0.0) durchgeführt.

Die in dieser Arbeit enthaltenen Abbildungen wurden mit Unterstützung von ChatGPT-4/5 erstellt.

## Ergebnisse

### Teilnehmermerkmale

An der Umfrage nahmen insgesamt 273 Ärztinnen und Ärzte aus allen Bundesländern teil, darunter 116 Männer (42,5 %) und 157 Frauen (57,5 %). Die Mehrheit (68 %) lebte in einer Partnerschaft, etwa die Hälfte (51,3 %) hatte Kinder (Tab. 1). Ein Drittel war zum Zeitpunkt der Befragung wissenschaftlich aktiv. Weitere 37 % hatten zwar Forschungsinteresse, waren jedoch nicht wissenschaftlich tätig; innerhalb dieser Gruppe waren Frauen mit 68 % deutlich überrepräsentiert. Weitere demografische Merkmale sowie Gruppenvergleiche sind in Tabelle S1 (Online-Supplement) dargestellt.

**Tab. 1:** Demografische Charakteristika der Stichprobe (*n* = 273).

*Geschlecht*	
Weiblich	157 (57,5)
männlich	116 (42,5)
*Alter*	
< 30 Jahre	18 (6,6)
30–39 Jahre	68 (24,9)
40–49 Jahre	90 (33,0)
50–59 Jahre	52 (19,0)
≥ 60 Jahre	45 (16,5)
*Familienstand*	
Verheiratet/Partnerschaft	185 (67,8)
Ledig	72 (26,4)
Keine Angabe	16 (5,9)
*Kinder*	
Ja	140 (51,3)
Nein	126 (46,2)
Keine Angabe	7 (2,6)

Angaben als *n* (%)

### Beruf und Karriere

Tab. 2 und Tabelle S2 (Zusatzmaterial online) geben einen Überblick über die aktuelle berufliche Situation und die angestrebten Karriereziele der Umfrageteilnehmenden. Wissenschaftlich Aktive waren mit 47 % überproportional häufig an Universitätskliniken mit geriatrischem Lehrstuhl tätig, obwohl nur 21 % der Gesamtkohorte an solchen Einrichtungen beschäftigt waren. Unter den aktuell wissenschaftlich nicht aktiven Teilnehmenden mit Forschungsinteresse arbeiteten 64 % an nichtuniversitären Kliniken ohne geriatrischen Lehrstuhl – dem häufigsten Arbeitgeber der Gesamtstichprobe (48 %). Erwartungsgemäß hatten wissenschaftlich Aktive häufiger eine Promotion oder Habilitation abgeschlossen und strebten wissenschaftliche Laufbahnen und klinische Führungspositionen, wie etwa Chefarztpositionen, an. Während über 40 % ihr klinisches Karriereziel als erreicht ansahen, hatten nur ca. 20 % ihr wissenschaftliches Karriereziel erreicht.

**Tab. 2:** Berufliche Situation und Karriereziele nach Forschungsstatus.

Anzahl der Teilnehmer	Gesamt, *n* = 273	Aktiv in Forschung, *n* = 91	Interesse, nicht aktiv, *n* = 101	Kein Interesse, *n* = 81	*p‑Wert*
*Klinische Erfahrung*	*–*				*0,048*
< 5 Jahre	41 (15,0)	14 (15,4)	14 (13,9)	13 (16,0)	–
5–9 Jahre	25 (9,2)	10 (11,0)	11 (10,9)	4 (4,9)	
10–19 Jahre	91 (33,3)	25 (27,5)	44 (43,6)	22 (27,2)	
20–29 Jahre	60 (22,0)	17 (18,7)	19 (18,8)	24 (29,6)	
> 30 Jahre	56 (20,5)	25 (27,5)	13 (12,8)	18 (22,2)	
*Arbeitsort*	*–*				*<* *0,001*
Uniklinik mit geriatrischem Lehrstuhl	57 (20,9)	43 (47,3)	10 (9,9)	4 (4,9)	–
Uniklinik ohne geriatrischen Lehrstuhl	12 (4,4)	5 (5,5)	6 (5,9)	1 (1,2)	
Nichtuniversitär mit geriatrischem Lehrstuhl	41(15,0)	11(12,1)	10 (9,9)	20 (24,7)	
Nichtuniversitär ohne geriatrischen Lehrstuhl	132 (48,4)	26 (28,6)	65 (64,4)	41 (50,6)	
Rehaklinik	12 (4,4)	1 (1,1)	6 (5,9)	5 (6,2)	
Praxis	18 (6,6)	4 (4,4)	10 (12,3)	4 (4,0)	
Sonstige	1 (0,4)	1 (1,1)	0 (0,0)	0 (0,0)	
*Anzahl der Betten*	*–*				*0,072*
< 20	28 (10,3)	12 (13,2)	11 (10,9)	5 (6,2)	–
20–49	90 (33,0)	24 (26,4)	42 (41,6)	24 (29,6)	
50–74	34 (12,5)	8 (8,8)	17 (16,8)	9 (11,1)	
75–100	56 (20,5)	22 (24,2)	14 (13,9)	20 (24,7)	
> 100	35 (12,8)	16 (17,6)	8 (7,9)	11 (13,6)	
Trifft nicht zu	30 (11,0)	9 (9,9)	9 (8,9)	12 (14,8)	
*Promoviert*	*–*				*<* *0,001*
Ja	166 (60,8)	78 (85,7)	48 (47,5)	40 (49,4)	–
Nein, aber in Arbeit	21 (7,7)	8 (8,8)	10 (9,9)	3 (3,7)	
Nein	86 (31,5)	5 (5,5)	43 (42,6)	38 (46,9)	
*Habilitiert*	*–*				*<* *0,001*
Ja	33 (12,1)	27 (29,7)	3 (3,0)	3 (3,7)	–
Nein, aber in Arbeit	29 (10,6)	27 (29,7)	2 (2,0)	0 (0,0)	
Nein	211 (77,3)	37 (40,7)	96 (95,0)	78 (96,3)	
*Klinisches Karriereziel*	*–*				
Facharzt	35 (12,8)	8 (8,8)	12 (14,8)	15 (14,9)	0,072
Oberarzt	58 (21,2)	24 (26,4)	20 (19,8)	14 (17,3)	0,331
Chefarzt	42 (15,4)	27 (29,7)	13 (12,9)	2 (2,5)	< 0,001
Praxis	11 (4,0)	2 (2,2)	3 (3,0)	6 (7,4)	0,205
Unentschlossen	34 (12,5)	3 (3,3)	22 (21,8)	9 (11,1)	< 0,001
Kein Ziel	9 (3,3)	1 (1,1)	40 (39,6)	4 (4,9)	0,332
Ziel bereits erreicht	114 (41,8)	33 (36,3)	40 (39,6)	41 (50,6)	0,140
*Wissenschaftliches Karriereziel*	*–*				
Promotion	75 (27,5)	20 (22,0)	38 (37,6)	17 (21,0)	0,016
Habilitation	64 (23,4)	49 (53,8)	14 (13,9)	1 (1,2)	< 0,001
Professur	24 (8,8)	22 (24,2)	2 (2,0)	0 (0,0)	< 0,001
Unentschlossen	43 (15,8)	5 (5,5)	32 (32,0)	6 (7,4)	< 0,001
Kein Ziel	74 (27,1)	3 (3,3)	18 (17,8)	53 (65,4)	< 0,001
Ziel bereits erreicht	53 (19,4)	25 (27,5)	12 (11,9)	16 (19,8)	0,024

Angaben als *n* (%), Prozentsätze spaltenbezogen. Fehlende Werte: wiss_Karriereziel_unentschlossen (*n* = 2)

### Wissenschaftliche Rahmenbedingungen, Interessen und Herausforderungen

Tabelle S3 im Online-Zusatzmaterial gibt einen Überblick zu Forschungsumfeld, individuellen Aktivitäten, Zeitmanagement, Interessen, Zufriedenheit und wahrgenommenen Hürden. Die große Mehrheit (71 %) der Teilnehmenden widmete weniger als 5 h/Woche der Forschung; lediglich 8 % investieren mehr als 20 h wöchentlich. Nur 21 % der Teilnehmerinnen und Teilnehmer gaben an, Forschungstätigkeiten während der regulären Arbeitszeit ausüben zu können.

**Abb. 1:**
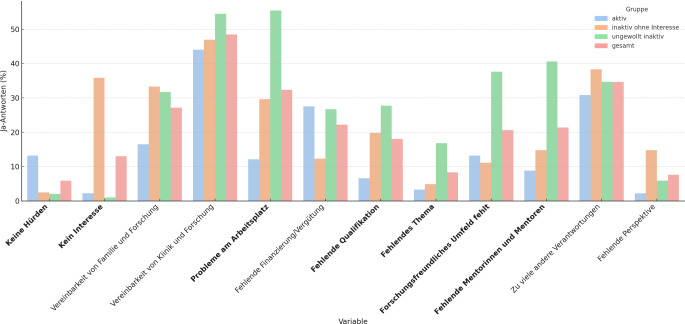
Nennung von Forschungshürden (in %) in der Gesamtstichprobe und nach Gruppen: wissenschaftlich aktiv, inaktiv mit Forschungsinteresse, ohne Forschungsinteresse. Signifikante Unterschiede fett hervorgehoben

Der größte Anteil der Befragten war unzufrieden mit den eigenen Forschungsmöglichkeiten. Die mangelnde Vereinbarkeit von klinischem Alltag und Forschung wurde von 49 % der Teilnehmenden als Hürde genannt und damit am häufigsten. In der Gruppe der Forschungsinteressierten, aber aktuell Nichtaktiven wurden Probleme am Arbeitsplatz mit 55 % am häufigsten genannt. Ebenfalls besonders häufig wurden das Fehlen von Mentoren (41 %) sowie ein wenig forschungsfreundliches Umfeld (38 %) angegeben. Abb. 1 zeigt die nach Forschungsstatus differenzierten Unterschiede bei den wahrgenommenen Forschungshürden.

### Geschlechterspezifische Unterschiede

**Abb. 2:**
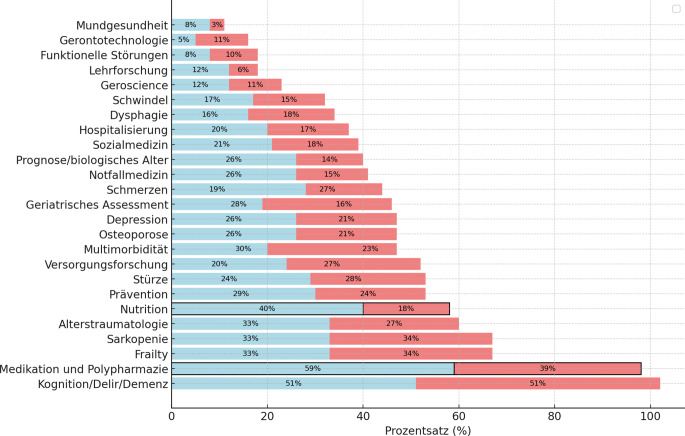
Nennung von relevanten Forschungsthemen (in %) durch männliche (*rot*) und weibliche (*blau*) Teilnehmer. Signifikante Geschlechterunterschiede *schwarz* *umrandet*

Eine geschlechtsspezifische Auswertung (Zusatzmaterial online: Tabelle S4) zeigte deutliche Unterschiede bei Alter, Qualifikation und wissenschaftlicher Beteiligung: Frauen waren tendenziell jünger, weniger häufig promoviert oder habilitiert und seltener wissenschaftlich aktiv als männliche Teilnehmer (26 % vs. 44 %). Männer verfügten öfter über einen geriatrischen Facharzttitel bzw. Zusatzweiterbildung (77 % vs. 59 %) und hatten häufiger Drittmittelerfahrung (32 % vs. 16 %). Geschlechterspezifische Unterschiede zeigten sich auch bei den Forschungsinteressen (Abb. 2): Frauen interessierten sich häufiger für Medikation/Polypharmazie (59 % vs. 39 %) und Ernährungsmedizin (40 % vs. 18 %) im Vergleich zu Männern.

### Rolle des Arbeitsumfelds

Ärzte an Universitätskliniken mit geriatrischem Lehrstuhl waren überdurchschnittlich oft promoviert (71 %) und habilitiert (24 %). Auch die Habilitation als Karriereziel wurde überwiegend an Universitätskliniken angegeben. Ärztinnen und Ärzte an nichtuniversitären Kliniken ohne assoziierten Lehrstuhl gaben häufig an, kein wissenschaftliches Karriereziel zu verfolgen (Zusatzmaterial online: Tabelle S5).

Obwohl die wissenschaftliche Beteiligung an nichtuniversitären Kliniken insgesamt geringer war, gaben 49 % der dort Tätigen an, trotz bislang fehlender Beteiligung an Forschungsprojekten interessiert zu sein.

Über alle Einrichtungen hinweg investierten die meisten Teilnehmenden < 5 h/Woche in Forschung. An Universitätskliniken waren es regelmäßig 5–10 h/Woche, an Universitätskliniken mit geriatrischem Lehrstuhl in einem von 5 Fällen sogar 20 h/Woche und mehr. Forschung erfolgte dort überproportional häufig sowohl während der Arbeitszeit als auch in der Freizeit. 19 % gaben geschützte Forschungszeiten an.

Die höchste Zufriedenheit mit den wissenschaftlichen Rahmenbedingungen bestand an Universitätskliniken mit geriatrischem Lehrstuhl (24 % sehr zufrieden, 26 % eher zufrieden). Die größte Unzufriedenheit berichteten Teilnehmer an nichtuniversitären Kliniken ohne Lehrstuhl (39 % eher oder sehr unzufrieden).

## Diskussion

Diese Umfrage liefert erstmals detaillierte Einblicke in die wissenschaftliche Beteiligung von Ärztinnen und Ärzten der Geriatrie in Deutschland sowie in deren demografische Struktur und berufliche Situation. Als zentrales Ergebnis zeigte sich, dass insbesondere Universitätskliniken mit Lehrstuhl für Geriatrie derzeit die besten Rahmenbedingungen für geriatrische Forschung bieten, was sich in der höchsten Promotions- und Habilitationsrate, der größten wissenschaftlichen Aktivität und der höchsten Zufriedenheit zeigt. Obschon wissenschaftliche Beteiligung an nichtuniversitären Einrichtungen seltener vorkommt, zeigte etwa die Hälfte der dort Beschäftigten Interesse an einer aktiven Mitarbeit in Forschungsprojekten.

Die Diskrepanz zwischen den häufig erreichten klinischen und den vergleichsweise selten erreichten wissenschaftlichen Karrierezielen deutet darauf hin, dass geriatrische Karrierewege in Deutschland bislang stark klinisch geprägt sind und wissenschaftliche Laufbahnen seltener sind. Dies korrespondiert mit der Wahrnehmung arbeitsplatzbezogener Probleme als zentraler Barriere für Forschungsengagement, was vermutlich auch auf ein mangelndes Verständnis für wissenschaftliche Arbeit in vielen Abteilungen und eine geringe institutionelle Unterstützung zurückzuführen ist.

Trotz des wachsenden Bedarfs an geriatrischer Expertise fehlen an vielen deutschen Universitätskliniken weiterhin geriatrische Lehrstühle, die Geriatern gezielte Forschungsförderung, Mentoring und wissenschaftlich Karrierewege und Netzwerkstrukturen ermöglichen, wie diese in andere Fachgebieten oftmals etabliert sind [13–15]. Nahezu alle medizinischen Fakultäten in Deutschland haben Clinician-Scientist-Programme implementiert, die geschützte Forschungszeit und strukturierte Weiterbildung bieten [7, 11] – auch für Geriaterinnen und Geriater, so denn eine geriatrische Abteilung vor Ort etabliert ist.

In anderen Fächern, wie etwa der Kardiologie, wurden die hohe Arbeitsbelastung und die mangelnde planbare Forschungszeit als zentrale Forschungshürden erkannt und konkrete Empfehlungen für ein nachhaltige Förderung ausgesprochen [16]. Eine Befragung geriatrischer Weiterbildungsassistenten in Großbritannien lieferte ähnliche Ergebnisse wie unsere Umfrage: 59 % der noch nicht in der Forschung tätigen Befragten äußerten den Wunsch, wissenschaftlich aktiv zu werden. Die Hälfte (51 %) nannte fehlende Mentoren und unzureichende Forschungszeit als zentrale Barrieren [17]. Internationale Untersuchungen zeigen, dass auch jenseits der Geriatrie klinisch tätige Ärztinnen und Ärzte mit wiederkehrenden Barrieren, die ihre Forschungsaktivitäten einschränken, konfrontiert sind. Zu den häufigsten Hürden zählen Zeitmangel, als unzureichend empfundene Ausbildung in Methodik und Statistik, konkurrierende klinische Verpflichtungen sowie ein Mangel an Anreizen und Anerkennung [18, 19]. Die hohe klinische Arbeitsbelastung verdrängt oft wissenschaftliche Ambitionen. Weltweit kämpfen Clinical Scientists zudem mit mangelndem Mentoring, unzureichender Finanzierung und der höheren Wertschätzung klinischer gegenüber wissenschaftlicher Tätigkeit [16, 17, 19, 20].

Geschlechtsspezifische Herausforderungen verschärfen die Problematik zusätzlich. Besonders problematisch erscheint, dass Frauen trotz großen Interesses an wissenschaftlicher Arbeit bislang offenbar deutlich weniger in Forschungsaktivitäten eingebunden sind. Internationale Erhebungen haben gezeigt, dass Ärztinnen im Zuge der Familiengründung häufig ihre Karrierewege anpassen müssen, was zu strukturell bedingten Ungleichheiten beiträgt [21]. Während Männer in den älteren Altersgruppen unserer Umfrage noch deutlich überrepräsentiert waren, waren Frauen in den jüngeren Altersgruppen deutlich in der Mehrheit, was auf eine künftig stärkere Prägung der Geriatrie durch Frauen hindeuten könnte. Insofern sind eine geschlechtersensible Perspektive und Strategien zur gezielten Förderung von Frauen in der geriatrischen Forschung besonders relevant [22]. Unsere Daten legen nahe, dass etwa Unterstützung bei Drittmittelanträgen und die Ermöglichung von Forschung während der Arbeitszeit zielgerichtet Unterstützung bieten könnten. Auch könnten von Frauen bevorzugte Themen gezielt für Förder- und Forschungsinitiativen genutzt werden.

Als generelle Lösungsansätze für die Förderung von Clinician Scientists werden in der Literatur wiederholt Maßnahmenbündel genannt, die sich auch in den in unserer Umfrage genannten Forschungshürden wiederspiegeln: geschützte Forschungszeit, die curriculare Verankerung von Forschungskompetenzen, der Ausbau von Mentoring-Netzwerken sowie gezielte Förderinitiativen [23–25]. In den USA wird der zunehmende Bedarf an spezialisierten Geriatern bereits mittels verschiedener Förderprogramme adressiert [20].

Insgesamt unterstreichen unsere Ergebnisse die Notwendigkeit gezielter Maßnahmen zur Förderung wissenschaftlicher Karrieren in der Geriatrie, um die akademische Entwicklung des Fachgebiets nachhaltig zu stärken: Neben allgemeinen Forderungen nach Mentoring, geschützten Forschungszeiten und dem Ausbau geriatrischer Lehrstühle, lassen sich konkrete Maßnahmen auf verschiedenen Ebenen benennen: Universitäten sollten geriatrische Lehrstühle mit klaren Forschungs- und Nachwuchsförderungsaufträgen ausstatten. Nichtuniversitäre Kliniken könnten Kooperationen mit Universitäten eingehen und mit projektbezogenen Freistellungen Forschung in ihren Reihen unterstützen. Nicht zuletzt können Fachgesellschaften eine Schlüsselrolle einnehmen bei der wissenschaftlichen Nachwuchsförderung, etwa durch Mentoring, Stipendien, methodische Workshops und die Sichtbarmachung erfolgreicher Karrierewege. Ebenso könnten Programme für Medizinstudierende frühzeitig auf eine akademische Laufbahn in der Geriatrie vorbereiten [25].

### Limitationen

Die Erhebung basiert auf Selbstauskünften, wodurch Verzerrungen durch soziale Erwünschtheit oder subjektive Selbsteinschätzungen möglich sind. Das Online-Format und die Rekrutierung über spezifische Fachkanäle könnten zu einer Überrepräsentation in der Fachcommunity vernetzter, wissenschaftlich interessierter und digital affiner Teilnehmender führen. Somit ist die Stichprobe möglicherweise nicht vollständig repräsentativ für alle geriatrisch tätigen Ärzte in Deutschland. Das Querschnittdesign erlaubt zudem nur korrelative Aussagen, keine kausalen Schlussfolgerungen.

### Fazit

Zusammenfassend verdeutlicht die Studie strukturelle und institutionelle Herausforderungen der geriatrischen Forschung in Deutschland. Um ein integratives, forschungsfreundliches Umfeld zu schaffen, sind gezielte Maßnahmen nötig, wie die Förderung der wissenschaftlichen Aktivität von Frauen, der Ausbau geriatrischer Lehrstühle, geschützte Forschungszeiten, organisatorische Unterstützung und Mentoringprogramme. Die Ergebnisse zeigen zugleich das hohe Interesse und Potenzial der in der Geriatrie tätigen Ärztinnen und Ärzte, das es zu nutzen gilt.
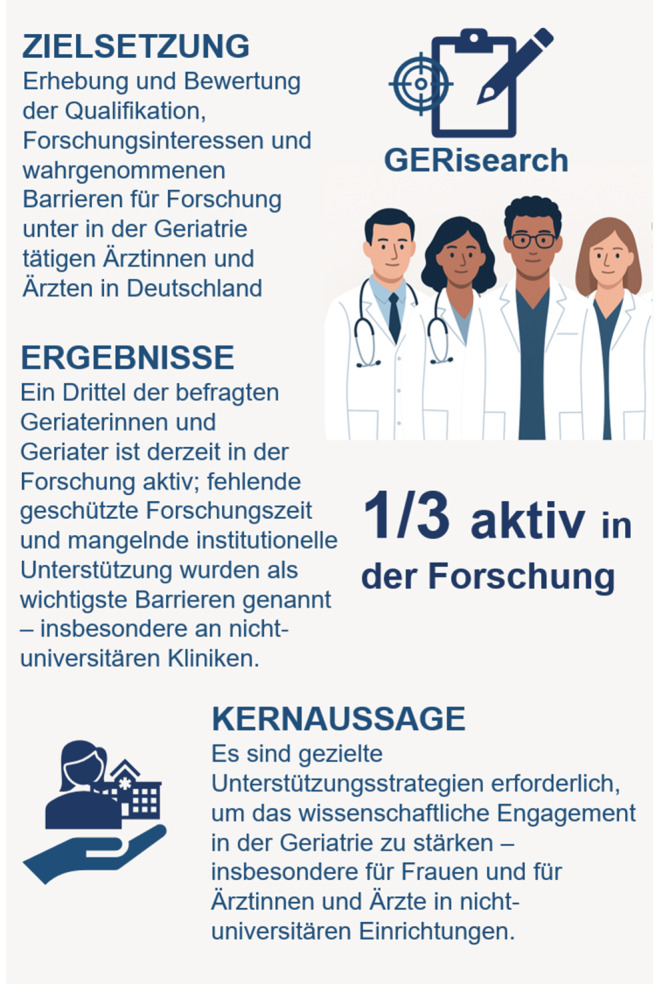


## Fazit für die Praxis

- Universitätskliniken mit geriatrischem Lehrstuhl bieten derzeit die besten Rahmenbedingungen für geriatrische Forschung; weitere Lehrstühle sind dringend erforderlich.
- Auch in nichtuniversitären Einrichtungen besteht ein großes Interesse an Forschungsprojekten. Das Potenzial zur Einbindung außeruniversitärer Geriaterinnen und Geriater – etwa im Rahmen wissenschaftlicher Kooperationen – sollte gezielt genutzt werden.
- Strukturelle Maßnahmen zur Förderung der geriatrischen Forschung sind erforderlich, insbesondere zur Unterstützung von Frauen in der Rolle von Clinician Scientists.
- Mentoringprogramme sollten etabliert werden, idealerweise direkt durch die Fachgesellschaften.

## Supplementary Information

Fragebogen

Tabellen S1–S5

## Data Availability

Die zugrunde liegenden Daten sind auf Anfrage beim korrespondierenden Autor verfügbar.
