# Supplementary material for: Challenges and potential of geriatric research in Germany—Insights from the GERisearch survey
Source: Z Gerontol Geriatr. 2025 Nov 26;59(2):132–9. [Article in German] doi: 10.1007/s00391-025-02528-z (PMC12953313; doi:10.1007/s00391-025-02528-z)
Supplement: Supplementary file 1 — Fragebogen [file 391_2025_2528_MOESM1_ESM.pdf]

Gemeinsame Umfrage

„Forschung in der Geriatrie/ Altersmedizin“

der AG Junge Geriatrie – Sektion Wissenschaft und der AG Wissenschaftsforum Geriatrie

Anna Maria Affeldt[1], Maela Caudal[2], Stefan Grund[3], Maximilian König[4], Bendix Labeit[5], Thea Laurentius[6], Varvara Moskiou[7], Johannes Trabert[8], Olaf Krause[9]

[1]Klinik II für Innere Medizin und Zentrum für Molekulare Medizin Köln, Universität zu Köln, Medizinische Fakultät und Universitätsklinikum Köln, Köln, Deutschland

[2]Altersmedizinisches Zentrum Köln, Cellitinnen-Krankenhaus St. Marien Köln, Deutschland

[3]Geriatrisches Zentrum am Universitätsklinikum Heidelberg, AGAPLESION Bethanien Krankenhaus Heidelberg, Heidelberg, Deutschland

[4]Klinik und Poliklinik für Innere Medizin D – Geriatrie, Universitätsmedizin Greifswald, Greifswald, Deutschland

[5]Klinik für Neurologie, Medizinische Fakultät, Heinrich-Heine-Universität Düsseldorf, Düsseldorf, Deutschland.

[6]Lehrstuhl für Geriatrie und Abteilung für Geriatrische Medizin, Uniklinik RWTH Aachen, Aachen, Deutschland

[7]Klinik für Geriatrie und Altersmedizin, Charité-Universitätsmedizin Berlin, Berlin, Deutschland

[8]AGAPLESION Markus Krankenhaus, Medizinisch-Geriatrische Klinik, Frankfurt, Deutschland

[9]Zentrum für Medizin im Alter, DIAKOVERE Henriettenstift, Hannover, Deutschland

Vielen Dank für Dein Interesse an unserer Umfrage „Forschung in der Geriatrie“.

Die Geriatrie ist eine Zukunfts- und Wachstumsdisziplin. Bislang gehört die Geriatrie/ Altersmedizin in Deutschland nicht zu den forschungsstarken Fächern. Über wissenschaftliche Qualifikationen, Interessen, potentielle Wünsche und Hindernisse in der Forschung von Geriater*innen ist wenig bekannt.

Mit dieser <u>anonymen</u> Umfrage möchten wir in kurzer Zeit (ca. 15 Minuten) Informationen über Erfahrungen, Interessen, Einstellungen und potentielle Hindernisse in Bezug auf wissenschaftliches Arbeiten in der Geriatrie/ Altersmedizin sammeln.

Vielen Dank, Eure AG Junge Geriatrie - Sektion Wissenschaft und AG Wissenschaftsforum Geriatrie der Deutschen Gesellschaft für Geriatrie (DGG)

Ich bin damit einverstanden, dass meine Antworten zu Forschungszwecken verwendet werden.

□ Ja

□ Nein

1. **Bist du approbierte/r Ärztin/ Arzt?**
   □ Ja
   □ Nein → *Dann kannst du leider nicht bei der Umfrage teilnehmen.*

2. **Geschlecht**
   □ weiblich
   □ männlich
   □ divers
   □ keine Angabe

3. **Alter**
   □ <30 Jahre
   □ 30-39 Jahre
   □ 40-49 Jahre
   □ 50-59 Jahre
   □ ≥60 Jahre

4. **In welchem Bundesland arbeitest du?**
   □ Baden-Württemberg
   □ Bayern
   □ Berlin
   □ Brandenburg
   □ Bremen
   □ Hamburg
   □ Hessen
   □ Mecklenburg-Vorpommern
   □ Niedersachsen
   □ Nordrhein-Westfalen
   □ Rheinland-Pfalz
   □ Saarland
   □ Sachsen-Anhalt
   □ Sachsen
   □ Schleswig-Holstein
   □ Thüringen
   □ Österreich
   □ Schweiz

5. **Familienstatus** (Mehrfachnennungen möglich)
   □ ledig
   □ verheiratet/ in einer Partnerschaft lebend
   □ Kind(er)
   □ keine Angabe

6. **Wie viele Jahre klinische Erfahrung in der Medizin nach dem Studium hast du?**
   □ <5 Jahre
   □ 5-9 Jahre
   □ 10-19 Jahre
   □ 20-29 Jahre
   □ ≥30 Jahre

7. **Welche Facharztbezeichnung/ Zusatzbezeichnung hast du?** (Mehrfachnennungen möglich)
   □ Keine
   □ Zusatzbezeichnung Geriatrie
   □ Innere Medizin
   □ Neurologie
   □ Innere Medizin und Geriatrie
   □ Orthopädie/ Unfallchirurgie
   □ Allgemeinmedizin
   □ Schwerpunkt-Internist (z.B. Kardiologie, Nephrologie, Gastroenterologie…)
   □ Rehabilitationsmedizin (Zusatzbezeichnung/ Facharzt)
   □ Sonstige: ____ *(Freitext)*

8. **Wo arbeitest du?** (Mehrfachnennungen möglich)
   □ Praxis
   □ Nicht-universitäres Krankenhaus <u>ohne</u> assoz. Lehrstuhl/Professur
   □ Nicht-universitäres Krankenhaus <u>mit</u> assoz. Lehrstuhl/Professur
   □ Uniklinik <u>mit</u> assoz. Lehrstuhl/Professur
   □ Uniklinik <u>ohne</u> assoz. Lehrstuhl/Professur
   □ Sonstiges: ____ *(Freitext)*

9. **Arbeitest du zur Zeit in einer geriatrischen Abteilung?**
   □ Ja
   □ Nein

10. Wie viele Betten stehen in deiner Abteilung zur Verfügung (ungefähr)?

□ <20

□ 20-49

□ 50-74

□ 75-100

□ >100

□ keine der Angaben trifft zu

11. Bist du promoviert/ habilitiert? (Mehrfachnennungen möglich)

□ nein, weder noch

□ promoviert

□ ich arbeite an meiner Promotion

□ habilitiert

□ ich arbeite an meiner Habilitation

12. Was ist dein angestrebtes <u>klinisches</u> Karriereziel in den nächsten 10 Jahren?
(Mehrfachnennungen möglich)

□ Facharzt/ Fachärztin

□ Oberarzt/ Oberärztin

□ Chefarzt/ Chefärztin

□ Niederlassung

□ Ich habe kein klinisches Karriereziel

□ Ich habe mein Ziel bereits erreicht

□ Ich weiß es noch nicht/ unentschlossen

□ Sonstiges: ____ *(Freitext)*

13. Was ist dein angestrebtes <u>wissenschaftliches</u> Karriereziel in den nächsten 10 Jahren?
(Mehrfachnennungen möglich)

□ Ich habe kein wissenschaftliches Karriereziel

□ Promotion

□ Habilitation

□ Arbeitsgruppenleitung

□ Lehrstuhl/ Professur

□ Ich habe mein Ziel bereits erreicht

□ Ich weiß es noch nicht/ unentschlossen

□ Sonstiges: ____ *(Freitext)*

14. Wird in deinem Arbeitsumfeld geriatrische/ altersmedizinische Forschung betrieben?

□ Ja, Forschung spielt eine wichtige Rolle

□ Ja, aber nur wenig/ punktuell

□ Nein, Forschung spielt keine Rolle

15. Bist du derzeit in der geriatrischen/ altersmedizinischen Forschung aktiv?

□ Ja

□ Nein, und ich möchte es auch nicht

□ Nein, aber ich wäre gerne in der Forschung tätig

□ Sonstiges: ____ *(Freitext)*

16. Wie würdest du deine Rolle in der Forschung beschreiben?

____ *(Freitext)*

17. Welche Forschungsthemen in der Geriatrie sind für dich am wichtigsten oder interessieren dich am meisten? (Mehrfachnennungen möglich)

□ Kognition (u.a. Delirium/ Demenz)

□ Gerontotechnologie

□ Medikation/ Polypharmazie

□ Geriatrisches Assessment

□ Alterstraumatologie

□ Stürze

□ Sozialmedizinische Fragestellungen

□ Prognose/ biologisches Alter

□ Hospitalisierung

□ Versorgungsforschung

□ Frailty

□ Sarkopenie

□ Nutrition

□ Forschung über Lehre in der Geriatrie

□ Multimorbidität

□ Oral health (Mundgesundheit/ Zahngesundheit)

□ Geroscience (Biologie des Alterns)

□ Prävention

□ Osteoporose

□ Depression im Alter

□ Dysphagie

□ Chronische Schmerzen

□ Geriatrische Notfallmedizin

□ Schwindel

□ Funktionelle Störungen

□ Sonstiges: ____ *(Freitext)*

18. Wie viele Stunden pro Woche arbeitest du in der Forschung (inkl. z.B. Freizeitforschung)?

□ <5 Stunden
□ 5-9 Stunden
□ 10-14 Stunden
□ 15-20 Stunden
□ >20 Stunden

19. Wann übst du deine Forschungstätigkeit hauptsächlich aus?

□ Während der Arbeitszeit, zusätzlich zur klinischen Tätigkeit
□ In der Freizeit, außerhalb der klinischen Tätigkeit.
□ Ich habe eine Beurlaubung/ geschützte Zeit für Forschungsarbeit, die auch eingehalten wird
□ Ich habe eine Beurlaubung/ geschützte Zeit für Forschungsarbeit, die nicht eingehalten wird
□ Sonstiges: ____ *(Freitext)*
□ Trifft nicht zu.

20. Hast du schon einmal einen Drittmittelantrag gestellt? (Mehrfachnennungen möglich)

□ Intramurale Drittmittel, erfolgreich
□ Intramurale Drittmittel, nicht erfolgreich
□ Öffentliche Gelder (DFG, BMBF, EU o.ä.), erfolgreich
□ Öffentliche Gelder (DFG, BMBF, EU o.ä.), nicht erfolgreich
□ Stiftungen, erfolgreich
□ Stiftungen, nicht erfolgreich
□ Industrie, erfolgreich
□ Industrie, nicht erfolgreich
□ Nein

21. Denkst du, dass die Geriatrie/ Altersmedizin ein Bereich ist, in dem Forschung wichtig ist?

□ Ja, und ich denke es wird genug geforscht
□ Ja, aber es wird noch zu wenig geforscht
□ Unentschlossen
□ Nein

22. **Wie zufrieden bist du insgesamt mit <u>deiner</u> derzeitigen wissenschaftlichen Tätigkeit in der Geriatrie?**

□ Sehr unzufrieden

□ Eher unzufrieden

□ Unentschieden - teilweise unzufrieden, teilweise zufrieden

□ Eher zufrieden

□ Sehr zufrieden

□ Trifft nicht zu

23. **Gibt es deiner Meinung nach Hindernisse, die speziell die Forschung in der Geriatrie/ Altersmedizin erschweren?**

□ Ja und ich schätze diese Problematik als relevant ein

□ Ja, aber ich schätze diese Problematik nicht als relevant ein

□ Unentschlossen

□ Nein

24. **Wenn ja, welche?** (Freitext)

25. **Bitte gib ggf. die Gründe an, warum du derzeit nicht/ wenig aktiv in der Forschung tätig bist.** (Mehrfachnennungen möglich)

□ Kein Interesse

□ Fehlende Vereinbarkeit von Familie und Forschungszeit

□ Fehlende Vereinbarkeit von klinischer Tätigkeit und Forschungszeit

□ Keine Möglichkeiten zu Forschen am Arbeitsplatz

□ Fehlende Finanzierung/ Vergütung

□ Ich fühle mich für die Forschung nicht qualifiziert genug

□ Ich habe bisher noch nicht das richtige Thema gefunden

□ Forschungsfreundliches Umfeld fehlt

□ Fehlende/r Mentor*in

□ Zu viele andere Verantwortungen

□ Fehlende Perspektive

□ Ich sehe nicht, dass ich einen relevanten Beitrag leisten könnte

□ Fehlende Möglichkeit Ergebnisse zu veröffentlichen

□ Psychologischer Druck/ Konkurrenzkampf

□ Es gibt keine Hürden

□ Sonstiges: ____

26. **Kannst du dir Lösungen für diese Hindernisse vorstellen? Wenn möglich, gib Beispiele an.**

____ *(Freitext)*
