# Supplementary material for: Challenges and potential of geriatric research in Germany—Insights from the GERisearch survey
Source: Z Gerontol Geriatr. 2025 Nov 26;59(2):132–9. [Article in German] doi: 10.1007/s00391-025-02528-z (PMC12953313; doi:10.1007/s00391-025-02528-z)
Supplement: Supplementary file 2 — Tabellen S1–S5 [file 391_2025_2528_MOESM2_ESM.docx]

**Geriatrische Forschung in Deutschland: Herausforderungen und Chancen – Erkenntnisse aus der GERisearch-Umfrage**

Online Supplement

**Tabelle S1.** Demografische Charakteristika der Gesamtstichprobe sowie nach wissenschaftlicher Aktivität und Forschungsinteresse stratifiziert

|  | |  | **Gesamt** | **Aktiv** | **Interesse, aber keine Aktivität** | **Kein Interesse** | **p-Wert** |
| --- | --- | --- | --- | --- | --- | --- | --- |
| n (%) | | | 273 (100,0) | 91 (33,3) | 101 (37,0) | 81 (29,7) |  |
| Geschlecht, n (%) | | |  |  |  |  | **0,003** |
|  | Männlich | | 117 (42,9) | 51 (56) | 32 (31,7) | 34 (42) |  |
|  | weiblich | | 156 (57,1) | 40 (44) | 69 (68,3) | 47 (58) |  |
| Alter, n (%) | | |  |  |  |  | 0,068 |
|  | < 30 Jahre | | 18 (6,6) | 9 (9,9) | 7 (6,9) | 2 (2,5) |  |
|  | 30-39 Jahre | | 68 (24,9) | 22 (24,2) | 26 (25,7) | 20 (24,7) |  |
|  | 40-49 Jahre | | 90 (33,0) | 26 (28,6) | 41 (40,6) | 23 (28,4) |  |
|  | 50- 59 Jahre | | 52 (19,0) | 17 (18,7) | 19 (18,8) | 16 (19,8) |  |
|  | > 60 Jahre | | 45 (16,5) | 17 (18,7) | 8 (7,9) | 20 (24,7) |  |
| Bundesland, n (%) | | |  |  |  |  | **<0,001** |
|  | Baden-Württemberg | | 46 (16,8) | 13 (14,3) | 20 (19,8) | 13 (16,0) |  |
|  | Bayern | | 64 (23,4) | 16 (17,6) | 22 (21,8) | 26 (32,1) |  |
|  | Berlin | | 14 (5,1) | 6 (6,6) | 7 (6,9) | 1 (1,2) |  |
|  | Brandenburg | | 6 (2,2) | 1 (1,1) | 3 (3,0) | 2 (2,5) |  |
|  | Bremen | | 3 (1,1) | 1 (1,1) | 2 (2,0) | 0 (0,0) |  |
|  | Hamburg | | 4 (1,5) | 3 (3,3) | 1 (1,0) | 0 (0,0) |  |
|  | Hessen | | 24 (8,8) | 6 (6,6) | 4 (4,0) | 14 (17,3) |  |
|  | Mecklenburg-Vorpommern | | 6 (2,2) | 6 (6,6) | 0 (0,0) | 0 (0,0) |  |
|  | Niedersachsen | | 12 (4,4) | 6 (6,6) | 3 (3,0) | 3 (3,7) |  |
|  | NRW | | 57 (20,9) | 24 (26,4) | 24 (23,8) | 9 (11,1) |  |
|  | Rheinland-Pfalz | | 7 (2,6) | 0 (0,0) | 4 (4,0) | 3 (3,7) |  |
|  | Saarland | | 2 (0,7) | 0 (0,0) | 0 (0,0) | 2 (2,5) |  |
|  | Sachsen-Anhalt | | 8 (2,9) | 4 (4,4) | 2 (2,0) | 2 (2,5) |  |
|  | Sachsen | | 5 (1,8) | 0 (0,0) | 4 (4,0) | 1 (1,2) |  |
|  | Schleswig-Holstein | | 7 (2,6) | 3 (3,3) | 1 (1,0 | 3 (3,7) |  |
|  | Thüringen | | 6 (2,2) | 0 (0,0) | 4 (4,0) | 2 (2,5) |  |
|  | Österreich | | 1 (0,4) | 1 (1,1) | 0 (0,0) | 0 (0,0) |  |
|  | Schweiz | | 1 (0,4) | 1 (1,1) | 0 (0,0) | 0 (0,0) |  |
|  | Neue Bundesländer | | 45 (16,5) | 17 (18,7) | 20 (19,8) | 8 (9,9) | 0,158 |
| Familienstand, n (%) | | |  |  |  |  | 0,832 |
|  | Verheiratet/verpartnert | | 185 (67,8) | 64 (70,3) | 64 (63,4) | 57 (70,4) |  |
|  | Ledig | | 72 (26,4) | 22 (24,2) | 30 (29,7) | 20 (24,7) |  |
|  | Keine Angabe | | 16 (5,9) | 5 (5,5) | 7 (6,9) | 4 (4,9) |  |
| Kinder, n (%) | | |  |  |  |  | 0,330 |
|  | Ja |  | 140 (51,3) | 41 (45,1) | 59 (58,4) | 40 (49,4) |  |
|  | Nein | | 126 (46,2) | 47 (51,6) | 41 (40,6) | 38 (46,9) |  |
|  | Keine Angabe | | 7 (2,6) | 3 (3,3) | 1 (1,0) | 3 (3,7) |  |

**Tabelle S2:** Berufliches Umfeld, Qualifikationen, Erfahrung und Karriereziele in der Gesamtstichprobe sowie nach wissenschaftlicher Aktivität und Forschungsinteresse stratifiziert.

|  | | **Gesamt** | **Aktiv** | **Interesse, aber keine Aktivität** | **Kein Interesse** | **p-Wert** |
| --- | --- | --- | --- | --- | --- | --- |
| n (%) | | 273 (100,0) | 91 (33,3) | 101 (37,0) | 81 (19,7) |  |
| Klinische Erfahrung, n (%) | |  |  |  |  | **0,048** |
|  | < 5 Jahre | 41 (15,0) | 14 (15,4) | 14 (13,9) | 13 (16,0) |  |
|  | 5 bis 9 Jahre | 25 (9,2) | 10 (11,0) | 11 (10,9) | 4 (4,9) |  |
|  | 10-19 Jahre | 91 (33,3) | 25 (27,5) | 44 (43,6) | 22 (27,2) |  |
|  | 20-29 Jahre | 60 (22,0) | 17 (18,7) | 19 (18,8) | 24 (29,6) |  |
|  | > 30 Jahre | 56 (20,5) | 25 (27,5) | 13 (23,2) | 18 (22,2) |  |
| Zusatzbezeichnung Geriatrie, n (%) | |  |  |  |  | 0,967 |
|  | Ja | 181 (66,3) | 61 (67,0) | 66 (65,3) | 54 (66,7) |  |
|  | Nein | 92(33,7) | 30 (33,0) | 35 (34,7) | 27 (33,3) |  |
| Facharzt, n (%) | |  |  |  |  | 0,378 |
|  | Innere Medizin | 147 (53,8) | 48 (52,7) | 58 (57,4) | 41 (50,6) |  |
|  | Neurologie | 35 (12,8) | 17 (18,7) | 11 (10,9) | 7 (8,6) |  |
|  | Allgemeinmedizin | 30 (11,0) | 5 (5,5) | 11 (10,9) | 14 (17,3) |  |
|  | Chirurgie | 5 (1,8) | 2 (2,2) | 1 (1,0) | 2 (2,5) |  |
|  | Sonstig | 8 (2,9) | 2 (2,2) | 3 (3,0) | 3 (3,7) |  |
|  | Keinen/in Weiterbildung | 48 (17,6) | 17 (18,7) | 17 (16,8) | 14 (17,3) |  |
| Arbeitsort, n (%) | |  |  |  |  | < **0,001** |
|  | Uniklinik mit ger. Lehrstuhl | 57 (20,9) | 43 (47,3) | 10 (9,9) | 4 (4,9) |  |
|  | Uniklinik ohne ger. Lehrstuhl | 12 (4,4) | 5 (5,5) | 6 (5,9) | 1 (1,2) |  |
|  | Nicht-universitär mit ger. Lehrstuhl | 41 (15,0) | 11 (12,1) | 10 (9,9) | 20 (24,7) |  |
|  | Nicht-universitärer ohne Lehrstuhl | 132 (48,4) | 26 (28,6) | 65 (64,4) | 41 (50,6) |  |
|  | Rehaklinik | 12 (4,4) | 1 (1,1) | 6 (5,9) | 5 (6,2) |  |
|  | Praxis | 18 (6,6) | 4 (4,4) | 10 (12,3) | 4 (4,0) |  |
|  | Sonstige | 1 (0,4) | 1 (1,1) | 0 (0,0) | 0 (0,0) |  |
| Anzahl Betten, n (%) | |  |  |  |  | 0,072 |
|  | < 20 | 28 (10,3) | 12 (13,2) | 11 (10,9) | 5 (6,2) |  |
|  | 20-50 | 90 (33,0) | 24 (26,4) | 42 (41,6) | 24 (29,6) |  |
|  | 50-75 | 34 (12,5) | 8 (8,8) | 17 (16,8) | 9 (11,1) |  |
|  | 75-100 | 56 (20,5) | 22 (24,2) | 14 (13,9) | 20 (24,7) |  |
|  | > 100 | 35 (12,8) | 16 (17,6) | 8 (7,9) | 11 (13,6) |  |
|  | Trifft nicht zu | 30 (11,0) | 9 (9,9) | 9 (8,9) | 12 (14,8) |  |
| Promoviert, n (%) | |  |  |  |  | **< 0,001** |
|  | Ja | 166 (60,8) | 78 (85,7) | 48 (47,5) | 40 (49,4) |  |
|  | Nein, aber in Arbeit | 21 (7,7) | 8 (8,8) | 10 (9,9) | 3 (3,7) |  |
|  | Nein | 86 (31,5) | 5 (5,5) | 43 (42,6) | 38 (46,9) |  |
| Habilitiert, n (%) | |  |  |  |  | **< 0,001** |
|  | Ja | 33 (12,1) | 27 (29,7) | 3 (3,0) | 3 (3,7) |  |
|  | Nein, aber in Arbeit | 29 (10,6) | 27 (29,7) | 2 (2,0) | 0 (0,0) |  |
|  | Nein | 211 (77,3) | 37 (40,7) | 96 (95,0) | 78 (96,3) |  |
| Klinisches Karriereziel , n (%) | |  |  |  |  |  |
|  | Facharzt/Fachärztin | 35 (12,8) | 8 (8,8) | 12 (14,8) | 15 (14,9) | 0,072 |
|  | Oberarzt/Oberärztin | 58 (21,2) | 24 (26,4) | 20 (19,8) | 14 (17,3) | 0,331 |
|  | Chefarzt/Chefärztin | 42 (15,4) | 27 (29,7) | 13 (12,9) | 2 (2,5) | **< 0,001** |
|  | Praxis | 11 (4,0) | 2 (2,2) | 3 (3,0) | 6 (7,4) | 0.205 |
|  | Unentschlossen | 34 (12,5) | 3 (3,3) | 22 (21,8) | 9 (1,1) | <**0,001** |
|  | Kein Ziel | 114 (41,8) | 33 (36,3) | 40 (39,6) | 41 (50,6) | 0,14 |
|  | Ziel bereits erreicht | 264 (96,7) | 90 (98,9) | 97 (96,0) | 77 (95,1) | 0,332 |
| Wissenschaftliches Karriereziel, n (%) | |  |  |  |  |  |
|  | Promotion | 75 (27,5) | 20 (22,0) | 38 (37,6) | 17 (21,0) | 0,016 |
|  | Habilitation | 64 (23,4) | 49 (53,8) | 14 (13,9) | 1 (1,2) | **< 0,001** |
|  | Professur | 24 (8,8) | 22 (24,2) | 2 (2,0) | 0 (0,0) | **< 0,001** |
|  | Unentschlossen | 43 (15,8) | 5 (5,5) | 32 (32,0) | 6 (7,4) | **< 0,001** |
|  | Kein Ziel | 74 (27,1) | 3 (3,3) | 18 (17,8) | 53 (65,4) | **< 0,001** |
|  | Ziel bereits erreicht | 53 (19,4) | 25 (27,5) | 12 (11,9) | 16 (19,8) | 0,024 |

**Tabelle S3.** Wissenschaftliche Rahmenbedingungen, Aktivitäten, Interessen, Zufriedenheit und Herausforderungen in der Gesamtstichprobe sowie nach wissenschaftlicher Aktivität und Forschungsinteresse stratifiziert.

|  | | | **Gesamt** | **Aktiv** | **Interesse, aber keine Aktivität** | **Kein Interesse** | **p-Wert** |
| --- | --- | --- | --- | --- | --- | --- | --- |
|  | n (%) |  | 273 (100,0) | 91 (33,3) | 101 (37,0) | 81 (19,7) |  |
| Forscht das Umfeld, n (%) | | |  |  |  |  | **< 0,001** |
|  | Ja, fester Bestandteil | | 79 (29,4) | 54 (60,0) | 14 (14,1) | 11 (13,8) |  |
|  | Ja, ein wenig | | 74 (27,5) | 31 (34,4) | 25 (25,3) | 18 (22,5) |  |
|  | Nein | | 116 (43,1) | 5 (5,6) | 60 (60,6) | 51 (63,8) |  |
| Wochenstunden in der Forschung, n (%) | | |  |  |  |  | **< 0,001** |
|  | < 5 | | 166 (70,9) | 29 (31,9) | 80 (93,0) | 57 (100,0) |  |
|  | 5 bis 9 | | 34 (14,5) | 29 (31,9) | 5 (5,8) | 0 (0,0) |  |
|  | 10 bis 14 | | 10 (4,3) | 9 (9,9) | 1 (1,2) | 0 (0,0) |  |
|  | 15 bis 20 | | 6 (2,6) | 6 (6,6) | 0 (0,0) | 0 (0,0) |  |
|  | > 20 | | 18 (7,7) | 18 (19,8) | 0 (0,0) | 0 (0,0) |  |
| Wann findet die Forschung statt, n (%) | | |  |  |  |  |  |
|  | Während der Arbeitszeit | | 57 (20,9) | 42 (46,2) | 11 (10,9) | 4 (4,9) | **< 0,001** |
|  | In der Freizeit | | 86 (31,5) | 58 (63,7) | 22 (21,8) | 6 (7,4) | **< 0,001** |
|  | Geschützte Forschungszeit | | 16 (5,9) | 15 (16,5) | 1 (6,3) | 0 (0,0) | **< 0,001** |
|  | Trifft nicht zu | | 130 (47,6) | 3 (3,3) | 66 (65,3) | 61 (75,3) | **< 0,001** |
| Forschungsinteressen, n (%) | | |  |  |  |  |  |
|  | Kognition/Delir/Demenz | | 140 (51,3) | 44 (48,4) | 52 (51,5) | 44 (54,3) | 0,736 |
|  | Gerontotechnologie | | 20 (7,3) | 10 (11,0) | 7 (6,9) | 3 (3,7) | 0,184 |
|  | Medikation und Polypharmazie | | 138 (50,5) | 33 (36,3) | 58 (57,4) | 47 (58,0) | 0,004 |
|  | Geriatrisches Assessment | | 61 (22,3) | 33 (36,3) | 17 (16,8) | 11 (13,6) | **< 0,001** |
|  | Alterstraumatologie | | 84 (30,8) | 19 (20,9) | 39 (38,6) | 26 (32,1) | 0,028 |
|  | Stürze | | 72 (26,4) | 23 (25,3) | 34 (33,7) | 15 (18,5) | 0,067 |
|  | Sozialmedizin | | 53 (19,4) | 13 (14,3) | 23 (22,8) | 17 (21,0) | 0,303 |
|  | Prognose/biologisches Alter | | 57 (20,9) | 16 (17,6) | 26 (25,7) | 15 (18,5) | 0,314 |
|  | Hospitalisierung | | 51 (18,7) | 10 (11,0) | 26 (25,7) | 15 (18,5) | 0,032 |
|  | Versorgungsforschung | | 69 (25,3) | 27 (29,7) | 29 (28,7) | 13 (16,0) | 0,074 |
|  | Frailty | | 90 (33,0) | 37 (40,7) | 28 (27,7) | 25 (30,9) | 0,145 |
|  | Sarkopenie | | 90 (33,0) | 37 (40,7) | 28 (27,7) | 25 (30,9) | 0,145 |
|  | Nutrition | | 83 (30,4) | 27 (29,7) | 39 (38,6) | 17 (21,0) | 0.036 |
|  | Lehrforschung | | 25 (9,2) | 12 (13,2) | 11 (10,9) | 2 (2,5) | 0,039 |
|  | Multimorbidität | | 79 (28,9) | 22 (24,2) | 33 (32,7) | 24 (29,6) | 0,426 |
|  | Mundgesundheit | | 17 (6,2) | 6 (6,6) | 5 (5,0) | 6 (7,4) | 0,78 |
|  | Geroscience | | 31 (11,4) | 12 (13,2) | 11 (10,9) | 8 (9,9) | 0,778 |
|  | Prävention | | 74 (27,1) | 21 (23,1) | 37 (36,6) | 16 (19,8) | 0,022 |
|  | Osteoporose | | 64 (23,4) | 21 (23,1) | 27 (26,7) | 16 (19,8) | 0,541 |
|  | Depression | | 64 (23,4) | 15 (16,5) | 26 (25,7) | 23 (28,4) | 0,145 |
|  | Dysphagie | | 46 (16,8) | 17 (18,7) | 19 (18,8) | 10 (12,3) | 0,434 |
|  | Schmerzen | | 63 (23,1) | 12 (13,2) | 27 (26,7) | 24 (29,6) | 0,021 |
|  | Notfallmedizin | | 58 (21,1) | 11 (12,1) | 30 (29,7) | 17 (21,0) | 0,012 |
|  | Schwindel | | 43 (15,8 | 10 (1,0) | 21 (20,8) | 12 (14,8) | 0,17 |
|  | Funktionelle Störungen | | 24 (8,8) | 3 (3,3) | 11 (10,9) | 10 (12,3) | 0,072 |
| Drittmittelanträge, n (%) | | |  |  |  |  |  |
|  | Erfolgreich, gesamt | | 58 (22,2) | 49 (53,8) | 5 (5,1) | 4 (5,6) | **< 0,001** |
|  | Noch nie gestellt | | 180 (69,0) | 27 (29,7) | 87 (88,8) | 66 (91,7) | **< 0,001** |
|  | Intramural, erfolgreich | | 28 (10,7) | 28 (38) | 0 (0,0) | 0 (0,0) | **< 0,001** |
|  | Intramural, nicht erfolgreich | | 17 (6,5) | 15 (16,5) | 2 (2,0) | 0 (0,0) | **< 0,001** |
|  | Öffentlich erfolgreich | | 34 (13,0) | 31 (34,1) | 1 (1,0) | 2 (2,8) | **< 0,001** |
|  | Öffentlich nicht erfolgreich | | 28 (10,7) | 22 (24,2) | 4 (4,1) | 2 (2,8) | **< 0,001** |
|  | Stiftung, erfolgreich | | 33 (12,6) | 25 (27,5) | 5 (5,1) | 3 (4,2) | **< 0,001** |
|  | Stiftung, nicht erfolgreich | | 17 (6,5) | 13 (14,3) | 2 (2,0) | 2 (2,8) | **< 0,001** |
|  | Industrie, erfolgreich | | 27 (10,3) | 21 (23,1) | 4 (4,1) | 2 (2,8) | **< 0,001** |
|  | Industrie, nicht erfolgreich | | 3 (1,1) | 3 (3,3) | 0 (0,0) | 0 (0,0) | 0.059 |
| Zufriedenheit mit wiss. Tätigkeit, n (%) | | |  |  |  |  | **< 0,001** |
|  | Sehr zufrieden | | 21 (8,0) | 17 (18,7) | 2 (2,0) | 2 (2,7) |  |
|  | Eher zufrieden | | 42 (15,9) | 30 (33,0) | 5 (5,1) | 7 (9,3) |  |
|  | Teils teils | | 44 (16,7) | 19 (20,9) | 20 (20,4) | 5 (6,7) |  |
|  | Eher unzufrieden | | 49 (18,6) | 16 (17,6) | 30 (30,6) | 3 (4,0) |  |
|  | Sehr unzufrieden | | 23 (8,7) | 7 (7,7) | 12 (12,2) | 4 (5,3) |  |
|  | Trifft nicht zu | | 85 (32,2) | 2 (2,2) | 29 (29,6) | 54 (72,0) |  |
| Hürden bei der Wissenschaft, n (%) | | |  |  |  |  |  |
|  | Keine Hindernisse | | 16 (5,9) | 12 (13,2) | 2 (2,0) | 2 (2,5) | **0,001** |
|  | Kein Interesse | | 32 (11,7) | 2 (2,2) | 1 (1,0) | 29(35,8) | **< 0,001** |
|  | Familie und Forschung | | 74 (27,1) | 15 (16,5) | 32 (31,7) | 27 (33,3) | 0,020 |
|  | Klinik und Forschung | | 133 (48,7) | 40 (44,0) | 55 (54,5) | 38 (46,9) | 0,323 |
|  | Probleme am Arbeitsplatz | | 91 (33,3) | 11 (12,1) | 56 (55,4) | 24 (29,6) | **< 0,001** |
|  | Fehlende Finanzierung/Vergütung | | 62 (22,7) | 25 (27,5) | 27 (26,7) | 10 (12,3) | 0,029 |
|  | Fehlende Qualifikation | | 50 (18,3) | 6 (6,6) | 28 (27,7) | 16 (19,8) | **< 0,001** |
|  | Fehlendes Thema | | 24 (8,8) | 3 (3,3) | 17 (16,8) | 4 (4,9) | **0,001** |
|  | Forschungsfreundliches Umfeld | | 59 (21,6) | 12 (13,2) | 38 (37,6) | 9 (11,1) | **< 0,001** |
|  | Fehlende MentorInnen | | 61 (22,3) | 8 (8,8) | 41 (40,6) | 12 (14,8) | **< 0,001** |
|  | Zu viele andere Verantwortungen | | 94 (34,4) | 28 (30,8) | 35 (34,7) | 31 (38,2) | 0,585 |
|  | Fehlende Perspektive | | 14 (5,1) | 4 (4,4) | 4 (4,0) | 6 (7,4) | 0,536 |
|  | Fehlen des eigenen Beitrags | | 20 (7,3) | 2 (2,2) | 6 (5,9) | 12 (14,8) | 0,005 |
|  | Sonstige Hürden | | 5 (1,8) | 1 (1,1) | 1 (1,0) | 3 (3,7) | 0, 325 |

**Tabelle S4.** Geschlechtsspezifisch stratifizierte Auswertung soziodemografischer Merkmale, Qualifikationen sowie wissenschaftlicher Aktivität, Hürden und Interessen.

|  | | | **Weiblich** | **Männlich** | **p-Wert** |
| --- | --- | --- | --- | --- | --- |
|  |  |  | n = 157 (57,5 %) | n = 116 (42,5 %) |  |
| Alter, n (%) | | |  |  | **<0,001** |
|  | < 30 Jahre | | 15 (10) | 3 (3) |  |
|  | 30-39 Jahre | | 41 (26) | 27 (23) |  |
|  | 40-40 Jahre | | 59 (38) | 31 (27) |  |
|  | 50-59 Jahre | | 31 (20) | 22 (19) |  |
|  | > 60 Jahre | | 11 (7) | 34 (29) |  |
| Bundesland, n (%) | | |  |  | 0,090 |
|  | Baden-Württemberg | | 23 (15) | 24 (20) |  |
|  | Bayern | | 39 (25) | 25 (21) |  |
|  | Berlin | | 5 (3) | 9 (8) |  |
|  | Brandenburg | | 4 (3) | 2 (2) |  |
|  | Bremen | | 2 (1) | 1 (1) |  |
|  | Hamburg | | 1 (1) | 3 (3) |  |
|  | Hessen | | 11 (7) | 13 (11) |  |
|  | Mecklenburg-Vorpommern | | 3 (2) | 3 (3) |  |
|  | Niedersachsen | | 10 (6) | 2 (2) |  |
|  | NRW | | 39 (25) | 18 (15) |  |
|  | Rheinland-Pfalz | | 2 (1) | 5 (4) |  |
|  | Saarland | | 0 (0) | 2 (2) |  |
|  | Sachsen-Anhalt | | 4 (3) | 4 (3) |  |
|  | Sachsen | | 4 (3) | 1 (1) |  |
|  | Schleswig-Holstein | | 5 (3) | 2 (2) |  |
|  | Thüringen | | 5 (3) | 1 (1) |  |
|  | Österreich | | 0 (0) | 1 (1) |  |
|  | Schweiz | | 0 (0) | 1 (1) |  |
|  | Neue Bundesländer gesamt | | 25 (16) | 20 (17) | 0,796 |
| Familienstand, n (%) | | |  |  | **0,002** |
|  | Verheiratet/verpartnert | | 93 (59) | 93 (80) |  |
|  | Ledig | | 53 (34) | 19 (16) |  |
|  | Keine Angabe | | 11 (7) | 5 (4) |  |
| Kinder, n (%) | | |  |  | 0,710 |
|  | Ja |  | 79 (50) | 62 (53) |  |
|  | Nein | | 73 (47) | 53 (45) |  |
|  | Keine Angabe | | 5 (3) | 2 (2) |  |
| Facharzt, n (%) | | |  |  | **0,038** |
|  | Innere Medizin | | 79 (50) | 69 (59) |  |
|  | Neurologie | | 15 (10) | 20 (15) |  |
|  | Allgemeinmedizin | | 23 (15) | 7 (6) |  |
|  | Chirurgie | | 2 (1) | 3 (3) |  |
|  | Sonstige | | 5 (3) | 3 (3) |  |
|  | In Weiterbildung | | 33 (21) | 15 (13) |  |
| Zusatzbezeichnung Geriatrie, n (%) | | |  |  | **<0,001** |
|  | Ja |  | 92 (59) | 90 (77) |  |
|  | Nein | | 65 (41) | 27 (23) |  |
| Klinische Erfahrung, n (%) | | |  |  | **<0,001** |
|  | < 5 Jahre | | 31 (20) | 10 (9) |  |
|  | 5 bis 9 Jahre | | 17 (11) | 8 (7) |  |
|  | 10-19 Jahre | | 57 (36) | 34 (29) |  |
|  | 20-29 Jahre | | 33 (21) | 28 (24) |  |
|  | > 30 Jahre | | 19 (12) | 37 (32) |  |
| Einrichtungstyp, n (%) | | |  |  | 0,831 |
|  | Uniklinik mit Lehrstuhl | | 36 (23) | 22 (19) |  |
|  | Uniklinik ohne Lehrstuhl | | 7 (5) | 5 (4) |  |
|  | Nicht-universitär mit Lehrstuhl | | 22 (14) | 19 (16) |  |
|  | Nicht-universitärer ohne Lehrstuhl | | 77 (49) | 55 (47) |  |
|  | Rehaklinik | | 6 (4) | 6 (5) |  |
|  | Praxis | | 9 (6) | 9 (8) |  |
| Anzahl Betten, n (%) | | |  |  | 0,200 |
|  | < 20 | | 16 (10) | 12 (10) |  |
|  | 20-50 | | 60 (38) | 30 (26) |  |
|  | 50-75 | | 21 (13) | 13 (11) |  |
|  | 75-100 | | 29 (19) | 27 (23) |  |
|  | > 100 | | 16 (10) | 20 (17) |  |
|  | Trifft nicht zu | | 15 (10) | 15 (13) |  |
| Promoviert, n (%) | | |  |  | 0,065 |
|  | Ja |  | 87 (55) | 80 (68) |  |
|  | Nein, aber in Arbeit | | 12 (8) | 9 (8) |  |
|  | Nein | | 58 (37) | 28 (24) |  |
| Habilitiert, n (%) | | |  |  | **0,006** |
|  | Ja |  | 11 (7) | 23 (20) |  |
|  | Nein, aber in Arbeit | | 16 (10) | 13 (11) |  |
|  | Nein | | 130 (83) | 81 (69) |  |
| Klinisches Karriereziel , n (%) | | |  |  |  |
|  | Facharzt/Fachärztin | | 29 (19) | 6 (5) | **0,001** |
|  | Oberarzt/Oberärztin | | 47 (30) | 11 (9) | **<0,001** |
|  | Chefarzt/Chefärztin | | 19 (12) | 23 (20) | 0,111 |
|  | Praxis | | 6 (4) | 5 (4) | 0,500 |
|  | Unentschlossen | | 22 (14) | 12 (10) | 0,351 |
|  | Kein Ziel | | 3 (2) | 6 (5) | 0,139 |
|  | Ziel bereits erreicht | | 55 (35) | 60 (51) | **0,007** |
| Wissenschaftliches Karriereziel, n (%) | | |  |  |  |
|  | Promotion | | 43 (27) | 32 (27) | 0,994 |
|  | Habilitation | | 32 (20) | 33 (28) | 0,231 |
|  | Professur | | 10 (6) | 14 (12) | 0,190 |
|  | Unentschlossen | | 32 (21) | 11 (9) | 0,013 |
|  | Kein Ziel | | 42 (27) | 32 (27) | 0,912 |
|  | Ziel bereits erreicht | | 27 (17) | 27 (23) | 0,226 |
| Wissenschaftlich aktiv, n (%) | | |  |  | **0,003** |
|  | Ja | | 40 (26) | 51 (44) |  |
|  | Nein, aber würde gerne aktiv sein | | 69 (44) | 32 (27) |  |
|  | Nein, kein Interesse | | 47 (30) | 34 (29) |  |
| Forscht das Umfeld, n (%) | | |  |  |  |
|  | Ja, fester Bestandteil | | 41 (27) | 38 (33) | 0,411 |
|  | Ja, ein wenig | | 41 (27) | 33 (29) |  |
|  | Nein | | 72 (47) | 44 (38) |  |
| Wochenstunden in der Forschung, n (%) | | |  |  | 0,064 |
|  | < 5 | | 101 (77) | 65 (63) |  |
|  | 5 bis 9 | | 12 (9) | 22 (21) |  |
|  | 10 bis 14 | | 6 (5) | 4 (4) |  |
|  | 15 bis 20 | | 2 (2) | 4 (4) |  |
|  | > 20 | | 10 (8) | 9 (9) |  |
| Wann findet die Forschung statt, n (%) | | |  |  |  |
|  | Während der Arbeitszeit | | 23 (15) | 34 (30) | **0,004** |
|  | In der Freizeit | | 41 (26) | 45 (39) | **0,029** |
|  | Geschützte Forschungszeit | | 8 (5) | 8 (7) | 0,543 |
|  | Trifft nicht zu | | 84 (54) | 47 (40) | **0,029** |
| Forschungsinteressen, n (%) | | |  |  |  |
|  | Kognition/Delir/Demenz | | 80 (51) | 60 (51) | 0,957 |
|  | Medikation und Polypharmazie | | 93 (59) | 45 (39) | **<0,001** |
|  | Frailty | | 51 (33) | 40 (34) | 0,767 |
|  | Sarkopenie | | 51 (33) | 40 (34) | 0,767 |
|  | Alterstraumatologie | | 52 (33) | 32 (27) | 0,305 |
|  | Nutrition | | 62 (40) | 21 (18) | **<0,001** |
|  | Multimorbidität | | 47 (20) | 32 (27) | 0,640 |
|  | Prävention | | 47 (30) | 27 (23) | 0,206 |
|  | Stürze | | 45 (29) | 28 (24) | 0,381 |
|  | Versorgungsforschung | | 37 (24) | 33 (28) | 0,384 |
|  | Osteoporose | | 40 (26) | 24 (21) | 0,337 |
|  | Depression | | 40 (26) | 24 (21) | 0,337 |
|  | Schmerzen | | 44 (28) | 19 (16) | 0,022 |
|  | Geriatrisches Assessment | | 29 (19) | 33 (27) | 0,057 |
|  | Notfallmedizin | | 40 (26) | 18 (15) | 0,043 |
|  | Prognose/biologisches Alter | | 41 (26) | 16 (14) | 0,012 |
|  | Sozialmedizin | | 33 (21) | 21 (18) | 0,527 |
|  | Hospitalisierung | | 31 (20) | 20 (17) | 0,577 |
|  | Dysphagie | | 25 (16) | 21 (18) | 0,657 |
|  | Schwindel | | 26 (17) | 17 (15) | 0,648 |
|  | Geroscience | | 18 (12) | 13 (11) | 0,927 |
|  | Lehrforschung | | 18 (12) | 7 (6) | 0,119 |
|  | Funktionelle Störungen | | 12 (8) | 12 (10) | 0,449 |
|  | Gerontotechnologie | | 7 (5) | 13 (11) | 0,036 |
|  | Mundgesundheit | | 13 (8) | 4 (3) | 0,099 |
| Drittmittelanträge, n (%) | | |  |  |  |
|  | Erfolgreich, gesamt | | 24 (16) | 35 (32) | **0,003** |
|  | Noch nie gestellt | | 115 (76) | 65 (59) | **0,002** |
|  | Intramural, erfolgreich | | 10 (7) | 18 (16) | 0,013 |
|  | Intramural, nicht erfolgreich | | 9 (6) | 8 (7) | 0,686 |
|  | Öffentlich erfolgreich | | 10 (7) | 25 (23) | **<0,001** |
|  | Öffentlich nicht erfolgreich | | 12 (8) | 16 (14) | 0,094 |
|  | Stiftung, erfolgreich | | 15 (10) | 18 (16) | 0,130 |
|  | Stiftung, nicht erfolgreich | | 6 (4) | 11 (10) | 0,054 |
|  | Industrie, erfolgreich | | 6 (4) | 21 (19) | **<0,001** |
|  | Industrie, nicht erfolgreich | | 1 (1) | 2 (2) | 0,392 |
| Zufriedenheit mit wiss. Tätigkeit, n (%) | | |  |  | 0,211 |
|  | Sehr zufrieden | | 10 (7) | 11 (10) |  |
|  | Eher zufrieden | | 20 (13) | 22 (20) |  |
|  | Teils teils | | 21 (14) | 23 (20) |  |
|  | Eher unzufrieden | | 30 (20) | 19 (17) |  |
|  | Sehr unzufrieden | | 16 (11) | 8 (7) |  |
|  | Trifft nicht zu | | 55 (36) | 30 (27) |  |
| Hürden bei der Wissenschaft, n (%) | | |  |  |  |
|  | Vereinbarkeit von Familie und Forschung | | 43 (27) | 31 (27) | 0,869 |
|  | Vereinbarkeit von Klinik und Forschung | | 83 (53) | 50 (43) | 0,097 |
|  | Probleme am Arbeitsplatz | | 58 (37) | 33 (28) | 0,129 |
|  | Fehlende Finanzierung/Vergütung | | 31 (20) | 31 (27) | 0,187 |
|  | Fehlende Qualifikation | | 36 (23) | 14 (12) | 0,020 |
|  | Fehlendes Thema | | 13 (8) | 11 (9) | 0,745 |
|  | Forschungsfreundliches Umfeld fehlt | | 39 (25) | 20 (17) | 0,123 |
|  | Fehlende Mentorinnen und Mentoren | | 37 (24) | 24 (21) | 0,548 |
|  | Zu viele andere Verantwortungen | | 52 (33) | 42 (36) | 0,632 |
|  | Fehlende Perspektive | | 7 (5) | 7 (6) | 0,571 |
|  | Fehlen des eigenen Beitrags | | 12 (8) | 8 (7) | 0,800 |
|  | Keine Hürden | | 6 (5) | 10 (9) | 0,165 |

***Anmerkungen:*** Werte sind als Anzahl und Prozent (Spaltenprozente) angegeben.

**Tabelle S5**. Nach Arbeitsort stratifizierte Analysen: soziodemografische Merkmale, Qualifikationen sowie wissenschaftliche Aktivitäten, Hürden und Interessen.

|  | | | **UK L+** | **UK L-** | **AKH L+** | **AKH L-** | **RK** | **Praxis** | **Sonstiges** | **p-Wert** |
| --- | --- | --- | --- | --- | --- | --- | --- | --- | --- | --- |
|  |  |  | n = 58 (21,2 %) | n = 12 (4,4 %) | n = 41 (15,0 %) | n = 132 (48,2 %) | n = 12 (4,4 %) | n = 18 (6,6 %) | n = 1  (0,4 %) |  |
| Alter, n (%) | | |  |  |  |  |  |  |  | **<0,001** |
|  | < 30 Jahre | | 9 (15,5) | 2 (16,7) | 1 (2,4) | 6 (4,5) | 0 (0.0) | 0 (0.0) | 0 (0,0) |  |
|  | 30-39 Jahre | | 20 (34,5) | 2 (16,7) | 21 (51,2) | 23 (17,4) | 2 (16,7) | 0 (0,0) | 0 (0,0) |  |
|  | 40-49 Jahre | | 19 (32,8) | 2 (16,7) | 6 (14,6) | 54 (40,9) | 4 (33,3) | 5 (27,8) | 0 (0,0) |  |
|  | 50- 59 Jahre | | 8 (13,8) | 6 (50,0) | 6 (14,6) | 22 (16,7) | 4 (33,3) | 7 (38,9) | 0 (0,0) |  |
|  | > 60 Jahre | | 2 (3,4) | 0 (0,0) | 7 (17,1) | 27 (20,5) | 2 (16,7) | 6 (33,3) | 1 (100,0) |  |
| Geschlecht, n (%) | | |  |  |  |  |  |  |  | 0,831 |
|  | Männlich | | 22 (37,9) | 5 (41,7) | 19 (46.3) | 55 (41,7) | 6 (50,0) | 9 (50,0) | 1 (100,0) |  |
|  | Weiblich | | 36 (62,1) | 7 (58,3) | 22 (53,7) | 77 (58,3) | 6 (50,0) | 9 (50,0) | 0 (0,0) |  |
| Bundesland, n (%) | | |  |  |  |  |  |  |  | **<0,001** |
|  | Baden-Württemberg | | 6 (10,3) | 0 (0,0) | 14 (34,1) | 21 (15,9) | 1 (8,3) | 5 (27,8) | 0 (0,0) |  |
|  | Bayern | | 8 (13,8) | 1 (8,3) | 10 (24,4) | 35 (26,5) | 6 (50,0) | 4(22,2) | 0 (0,0) |  |
|  | Berlin | | 6 (10,3) | 2 (16,7) | 0 (0,0) | 4 (3,0) | 0 (0,0) | 2 (11,1) | 0 (0,0) |  |
|  | Brandenburg | | 0 (0,0) | 3 (25,0) | 0 (0,0) | 2 (1,5) | 1 (8,3) | 0 (0,0) | 0 (0,0) |  |
|  | Bremen | | 0 (0,0) | 0 (0,0) | 0 (0,0) | 3 (2,3) | 0 (0,0) | 0 (0,0) | 0 (0,0) |  |
|  | Hamburg | | 1 (1,7) | 0 (0,0) | 0 (0,0) | 3 (2,3) | 0 (0,0) | 0 (0,0) | 0 (0,0) |  |
|  | Hessen | | 0 (0,0) | 0 (0,0) | 8 (19,5) | 15 (11,4) | 0 (0,0) | 0 (0,0) | 1 (100,0) |  |
|  | Mecklenburg-Vorpommern | | 5 (8,6) | 0 (0,0) | 1 (2,4) | 0 (0,0) | 0 (0,0) | 0 (0,0) | 0 (0,0) |  |
|  | Niedersachsen | | 4 (6,9) | 0 (0,0) | 1 (2,4) | 5 (3,8) | 0 (0,0) | 2 (11,1) | 0 (0,0) |  |
|  | NRW | | 22 (37,9) | 2 (16,7) | 5 (12,2) | 21 (15,9) | 3 (25,0) | 4 (22,2) | 0 (0,0) |  |
|  | Rheinland-Pfalz | | 0 (0,0) | 0 (0,0) | 1 (2,4) | 4 (3,0) | 1 (8,3) | 1 (5,6) | 0 (0,0) |  |
|  | Saarland | | 0 (0,0) | 0 (0,0) | 0 (0,0) | 2 (1,5) | 0 (0,0) | 0 (0,0) | 0 (0,0) |  |
|  | Sachsen-Anhalt | | 1 (1,7) | 0 (0,0) | 1 (2,4) | 6 (4,5) | 0 (0,0) | 0 (0,0) | 0 (0,0) |  |
|  | Sachsen | | 0 (0,0) | 0 (0,0) | 0 (0,0) | 5 (3,8) | 0 (0,0) | 0 (0,0) | 0 (0,0) |  |
|  | Schleswig-Holstein | | 1 (1,7) | 1 (8,3) | 0 (0,0) | 5 (3,8) | 0 (0,0) | 0 (0,0) | 0 (0,0) |  |
|  | Thüringen | | 3 (5,2) | 2 (16,7) | 0 (0,0) | 1 (0,8) | 0 (0,0) | 0 (0,0) | 0 (0,0) |  |
|  | Österreich | | 0 (0,0) | 1 (8,3) | 0 (0,0) | 0 (0,0) | 0 (0,0) | 0 (0,0) | 0 (0,0) |  |
|  | Schweiz | | 1 (1,7) | 0 (0,0) | 0 (0,0) | 0 (0,0) | 0 (0,0) | 0 (0,0) | 0 (0,0) |  |
| Familienstand, n (%) | | |  |  |  |  |  |  |  | 0,399 |
|  | Verheiratet/verpartnert | | 40 (69,0) | 7 (58,3) | 24 (58,5) | 95 (72,0) | 6 (50,0) | 14 (77,8) | 0 (0,0) |  |
|  | Ledig | | 17 (29,3) | 4 (33,3) | 14 (34,1) | 29 (22,0) | 5 (41,7) | 2 (11,1) | 1 (100,0) |  |
|  | Keine Angabe | | 1 (1,7) | 1 (8,3) | 3 (7,3) | 8 (6,1) | 1 (8,3) | 2 (11,1) | 0 (0,0) |  |
| Kinder, n (%) | | |  |  |  |  |  |  |  | 0,37 |
|  | Ja |  | 26 (44,8) | 6 (50,0) | 16 (39,0) | 73 (55,3) | 6 (50,0) | 13 (72,2) | 1 (100,0) |  |
|  | Nein | | 31 (53,4) | 5 (41,7) | 23 (56,1) | 57 (43,2) | 5 (41,7) | 5 (27,8) | 0 (0,0) |  |
|  | Keine Angabe | | 1 (1,7) | 1 (8,3) | 2 (4,9) | 2 (1,5) | 1 (8,3) | 0 (0,0) | 0 (0,0) |  |
| Klinische Erfahrung, n (%) | | |  |  |  |  |  |  |  | **<0,001** |
|  | < 5 Jahre | | 16 (27,6) | 2 (16,7) | 8 (19.5) | 15 (11,4) | 0 (0,0) | 0 (0,0) | 0 (0,0) |  |
|  | 5 bis 9 Jahre | | 7 (12,1) | 1 (8,3) | 9 (22,0) | 5 (3,8) | 2 (16,7) | 1 (5,6) | 0 (0,0) |  |
|  | 10-19 Jahre | | 22 (37,9) | 5 (41,7) | 10 (24,4) | 46 (34,8) | 4 (33,3) | 4 (22,0) | 0 (0,0) |  |
|  | 20-29 Jahre | | 7 (12,1%) | 3 (25,0) | 7 (17,1) | 34 (25,8) | 4 (33,3) | 6 (33,3) | 0 (0,0) |  |
|  | > 30 Jahre | | 6 (10,3) | 1 (8,3) | 7 (17,1) | 32 (24,2) | 2 (16,7) | 7 (38,9) | 1 (100,0) |  |
| Zusatzbezeichnung Geriatrie, n (%) | | |  |  |  |  |  |  |  | **<0,001** |
|  | Ja |  | 28 (48,3) | 6 (50,0) | 23 (56,1) | 101 (76,5) | 11(91,7) | 13 (72,2) | 0 (0,0) |  |
|  | Nein | | 30 (51,7) | 6 (50,0) | 18 (43,9) | 31 (23,5) | 1 (8,3) | 5 (27,8) | 1 (100,0) |  |
| Facharzt, n (%) | | |  |  |  |  |  |  |  | **<0,001** |
|  | Innere Medizin | | 29 (50,0) | 9 (75,0) | 17 (41,5) | 80 (60,6) | 3 (25,0) | 10 (55,6) | 0 (0,0) |  |
|  | Neurologie | | 6 (10,3) | 0 (0,0) | 6 (14,6) | 19 (14,4) | 0 (0.0) | 3 (16,7) | 1 (100,0) |  |
|  | Allgemeinmedizin | | 1 (1,7) | 1(3.3) | 5 (12,2) | 11 (8,3) | 7 (58,3) | 5 (27,8) | 0 (0,0) |  |
|  | Chirurgie | | 1 (1,7) | 0 (0,0) | 2 (4,9) | 2 (1,5) | 0 (0,0) | 0 (0,0) | 0 (0,0) |  |
|  | Sonstig | | 0 (0,0) | 0 (0,0) | 1 (2,4) | 6 (4,5) | 1 (8,3) | 0 (0,0) | 0 (0,0) |  |
|  | Keinen/in Weiterbildung | | 21 (36,2) | 2 (16,7) | 10 (24,4) | 14 (10,6) | 1 (8,3) | 0 (0,0) | 0 (0,0) |  |
| Anzahl Betten, n (%) | | |  |  |  |  |  |  |  | **<0,001** |
|  | < 20 | | 9 (15,5) | 2 (16,7) | 1 (2,4) | 16 (12,1) | 0 (0,0) | 0 (0,0) | 0 (0,0) |  |
|  | 20-50 | | 20 (34,5) | 5 (41,7) | 7 (17,1) | 53 (40,2) | 4 (33,3) | 1 (5,6) | 0 (0,0) |  |
|  | 50-75 | | 4 (6,9) | 0 (0,0) | 8 (19,5) | 19 (14,4) | 3 (25,0) | 0 (0,0) | 0 (0,0) |  |
|  | 75-100 | | 15 (25,9) | 3 (25,0) | 15 (36,6) | 20 (15,2) | 3 (25,0) | 0 (0,0) | 0 (0,0) |  |
|  | > 100 | | 7 (12,1) | 0 (0,0) | 9 (22,0) | 19 (14,4) | 1 (8,3) | 0 (0,0) | 0 (0,0) |  |
|  | Trifft nicht zu | | 3 (5,2) | 2 (16,7) | 1 (2,4) | 5 (3,8) | 1 (8,3) | 17 (94,4) | 1 (100,0) |  |
| Promoviert, n (%) | | |  |  |  |  |  |  |  | **0,012** |
|  | Ja |  | 41(70,7) | 8 (66,7) | 20 (48,8) | 81 (61,4) | 5 (41,7) | 11 (61,1) | 1 (100,0) |  |
|  | Nein, aber in Arbeit | | 7 (12,1) | 3 (25,0) | 5 (12,2) | 4 (3,0) | 0 (0,0) | 2 (11,1) | 0 (0,0) |  |
|  | Nein | | 10 (17,2) | 1 (8,3) | 16 (39,0) | 47 (35,6) | 7 (58,3) | 5 (27,8) | 0 (0,0) |  |
| Habilitiert, n (%) | | |  |  |  |  |  |  |  | **<0,001** |
|  | Ja |  | 14 (24,1) | 1 (8,3) | 4 (9,8) | 11 (8,3) | 0 (0,0) | 3 (16,7) | 1 (100,0) |  |
|  | Nein, aber in Arbeit | | 21 (36,2) | 1 (8,3) | 1 (2,4) | 6 (4,5) | 0 (0,0) | 0 (0,0) | 0 (0,0) |  |
|  | Nein | | 23 (39,7) | 10 (83,3) | 36 (87,8) | 115 (87,1) | 12 (100,0) | 15 (83,3) | 0 (0,0) |  |
| Klinisches Karriereziel , n (%) | | |  |  |  |  |  |  |  |  |
|  | Facharzt/Fachärztin | | 14 (24,1) | 2 (16,7) | 5 (12,2) | 14 (10,6) | 0 (0,0) | 0 (0,0) | 0 (0,0) | 0,178 |
|  | Oberarzt/Oberärztin | | 18 (31.0) | 3 (25,0) | 9 (22,0) | 26 (19,7) | 2 (16,7) | 0 (0,0) | 0 (0,0) | 0,618 |
|  | Chefarzt/Chefärztin | | 13 (22,4) | 1 (8,3) | 5 (12,2) | 23 (17,4) | 0 (0,0) | 0 (0,0) | 0 (0,0) | 0,598 |
|  | Praxis | | 3 (5,2) | 1 (8,3) | 2 (4,9) | 3 (2,3) | 0 (0,0) | 2 (11,1) | 0 (0,0) | 0,924 |
|  | Unentschlossen | | 8 (13,8) | 2 (16,7) | 8 (19,5) | 12 (9,1) | 1 (8,3) | 3 (16,7) | 0 (0,0) | 0,654 |
|  | Kein Ziel | | 1 (1,7) | 0 (0,0) | 3 (7,3) | 5 (3,8) | 0 (0,0) | 0 (0,0) | 0 (0,0) | 0,662 |
|  | Ziel bereits erreicht | | 13 (22,4) | 5 (41,7) | 12 (29,3) | 62 (47,0) | 8 (66,7) | 14 (77,8) | 1 (100,0) | **<0,001** |
| Wiss. Karriereziel, n (%) | | |  |  |  |  |  |  |  |  |
|  | Promotion | | 10 (17,2) | 4 (33,3) | 14 (34,1) | 37 (28,0) | 2 (16,7) | 8 (44,4) | 0 (0,0) | 0,246 |
|  | Habilitation | | 31 (53,4) | 5 (41,7) | 7 (17,1) | 18 (13,6) | 1 (8,3) | 2 (11,1) | 1 (100,0) | **<0,001** |
|  | Professur | | 12 (20,7) | 1 (8,3) | 4 (9,8) | 7 (5,3) | 0 (0,0) | 0 (0,0) | 0 (0,0) | 0,176 |
|  | Unentschlossen | | 6 (10,3) | 3 (25,0) | 11 (26,8) | 18 (13,7) | 3 (25,0) | 2 (11,1) | 0 (0,0) | 0,278 |
|  | Kein Ziel | | 5 (8,6) | 1 (8,3) | 11 (26,8) | 48 (36,4) | 5 (41,7) | 4 (22,2) | 0 (0,0) | 0,003 |
|  | Ziel bereits erreicht | | 12 (20,7) | 1 (8,3) | 6 (14,6) | 22 (16,7) | 1 (8,3) | 11 (61,1) | 1 (100,0) | **<0,001** |
| Wissenschaftlich aktiv, n (%) | | |  |  |  |  |  |  |  | **< 0,001** |
|  | Ja |  | 43 (75,4) | 5 (41,7) | 11 (26,8) | 26 (19,7) | 1 (8,3) | 4 (22,2) | 1 (100,0) |  |
|  | Nein, aber würde gerne aktiv sein | | 10 (17,5) | 6 (50,0) | 10 (24,4) | 65 (49,2) | 6 (50,0) | 4 (22,2) | 0 (0,0) |  |
|  | Nein, kein Interesse | | 4 (7,0) | 1 (8,3) | 20 (48,8) | 41 (31,1) | 5 (41,7) | 10 (55,6) | 0 (0,0) |  |
| Forscht das Umfeld, n (%) | | |  |  |  |  |  |  |  | **<0,001** |
|  | Ja, fester Bestandteil | | 45 (78,9) | 2 (16,7) | 20 (48,8) | 8 (6,1) | 2 (16,7) | 1 (6,3) | 1 (100,0) |  |
|  | Ja, ein wenig | | 11 (19,3) | 7 (58,3) | 9 (22,0) | 41 (31,3) | 2 (16,7) | 4 (25,0) | 0 (0,0) |  |
|  | Nein | | 1 (1,8) | 3 (25,0) | 12 (29,3) | 81 (61,8) | 8 (66,7) | 11 (68,8) | 0 (0,0) |  |
| Wochenstunden in der Forschung, n (%) | | |  |  |  |  |  |  |  | **<0,001** |
|  | < 5 | | 21 (37,5) | 6 (54,5) | 25 (78,1) | 94 (84,7) | 8 (88,9) | 12 (80,0) | 0 (0,0) |  |
|  | 5 bis 9 | | 11 (19,6) | 3 (27,3) | 2 (6,3) | 14 (12,6) | 1 (11,1) | 3 (20,0) | 0 (0,0) |  |
|  | 10 bis 14 | | 8 (14,3) | 1 (9,1) | 0 (0,0) | 1 (0,9) | 0 (0,0) | 0 (0,0) | 0 (0,0) |  |
|  | 15 bis 20 | | 5 (8,9) | 0 (0,0) | 1 (3,1) | 0 (0,0) | 0 (0,0) | 0 (0,0) | 0 (0,0) |  |
|  | > 20 | | 11 (19,6) | 1 (9,1) | 4 (12,5) | 2 (1,8) | 0 (0,0) | 0 (0,0) | 1 (100,0) |  |
| Wann findet die Forschung statt, n (%) | | |  |  |  |  |  |  |  |  |
|  | Während der Arbeitszeit | | 23 (39,7) | 4 (33,3) | 8 (19,5) | 19 (14,4) | 1 (8,3) | 2 (11,1) | 0 (0,0) | **0,004** |
|  | In der Freizeit | | 31 (53,4) | 4 (33,3) | 12 (29,3) | 31 (23,5) | 3 (25,0) | 5 (27,8) | 0 (0,0) | **0,007** |
|  | Geschützte Forschungszeit | | 11 (19,0) | 0 (0,0) | 4 (9,8) | 1 (0,8) | 0 (0,0) | 0 (0,0) | 0 (0,0) | **<0,001** |
|  | Trifft nicht zu | | 10 (17,2) | 4 (33,3) | 17 (41,5) | 78 (59,1) | 10 (83,3) | 12 (66,7) | 0 (0,0) | **<0,001** |
| Forschungsinteressen, n (%) | | |  |  |  |  |  |  |  |  |
|  | Kognition/Delir/Demenz | | 24 (41,4) | 6 (50,0) | 22 (53,7) | 70 (53,0) | 7 (58,3) | 11 (61,1) | 0 (0,0) | 0,606 |
|  | Gerontotechnologie | | 3 (5,2) | 2 (16,7) | 6 (14,6) | 6 (4,5) | 2 (16,7) | 1 (5,6) | 0 (0,0) | 0,21 |
|  | Medikation und Polypharmazie | | 24 (41,4) | 8 (66,7) | 17 (41,5) | 75 (54,8) | 8 (66,7) | 6 (33,3) | 0 (0,0) | 0,088 |
|  | Geriatrisches Assessment | | 23 (39,7) | 4 (33,3) | 6 (14,6) | 23 (17,4) | 2 (16,7) | 4 (22,2) | 0 (0,0) | 0,025 |
|  | Alterstraumatologie | | 14 (24,1) | 3 (25,0) | 6 (14,6) | 54 (40,9) | 3 (25,0) | 4 (22,2) | 0 (0,0) | 0,029 |
|  | Stürze | | 18 (31,0) | 4 (33,3) | 9 (22,0) | 35 (26,5) | 5 (41,7) | 2 (11,1) | 0 (0,0) | 0,508 |
|  | Sozialmedizin | | 7 (12,1) | 4 (33,3) | 13 (31,7) | 19 (14,4) | 3 (25,0) | 8 (44,4) | 0 (0,0) | 0,009 |
|  | Prognose/biologisches Alter | | 14 (24,1) | 3 (25,0) | 10 (24,4) | 24 (18,2) | 3 (25,0) | 3 (16,7) | 0 (0,0) | 0,923 |
|  | Hospitalisierung | | 8 (13,8) | 5 (41,7) | 9 (22,0) | 21 (15,9) | 3 (25,0) | 5 (27,8) | 0 (0,0) | 0,27 |
|  | Versorgungsforschung | | 13 (22,4) | 5 (41,7) | 12(29,3) | 28 (21,2) | 1 (8,3) | 10 (55,6) | 1 (100,0) | 0,01 |
|  | Frailty | | 29 (50,0) | 4 (33,3) | 13 (31,7) | 38 (28,8) | 4 (33,3) | 3 (16,7) | 0 (0,0) | 0,08 |
|  | Sarkopenie | | 29 (50,0) | 4 (33,3) | 13 (31,7) | 38 (28,8) | 4 (33,3) | 3 (16,7) | 0 (0,0) | 0,08 |
|  | Nutrition | | 23 (39,7) | 3 (25,0) | 10 (24,4) | 41 (31,1) | 3 (25,0) | 3 (16,7) | 0 (0,0) | 0,487 |
|  | Lehrforschung | | 10 (17,2) | 3 (25,0) | 1 (2,4) | 9 (6,8) | 1 (8,3) | 1 (5,6) | 0 (0,0) | 0,069 |
|  | Multimorbidität | | 15 (25,9) | 3 (25,0) | 14 (34,1) | 36 (27,3) | 2 (16,7) | 9 (50,0) | 0 (0,0) | 0,395 |
|  | Mundgesundheit | | 3 (5,2) | 0 (0,0) | 3 (7,3) | 8 (6,1) | 0 (0,0) | 3 (16,7) | 0 (0,0) | 0,514 |
|  | Geroscience | | 10 (17,2) | 3 (25,0) | 3 (7,3) | 12 (9,1) | 1 (8,3) | 2 (11,1) | 0 (0,0) | 0,445 |
|  | Prävention | | 15 (25,9) | 4 (33,3) | 12 (29,3) | 30 (22,7) | 4 (33,3) | 9 (50,0) | 0 (0,0) | 0,316 |
|  | Osteoporose | | 15 (25,9) | 3 (25,0) | 9 (22,0) | 31 (23,5) | 4 (33,3) | 2 (11,1) | 0 (0,0) | 0,84 |
|  | Depression | | 12 (20,7) | 4 (33,3) | 9 (22,0) | 28 (21,1) | 3 (25,0) | 7 (38,9) | 1 (100,0) | 0,32 |
|  | Dysphagie | | 10 (17,2) | 3 (25,0) | 5 (12,2) | 25 (18,9) | 1 (8,3) | 2 (11,1) | 0 (0,0) | 0,824 |
|  | Schmerzen | | 9 (15,1) | 3 (25,0) | 8 (19,5) | 32 (24,2) | 4 (33,3) | 7 (38,9) | 0 (0,0) | 0,441 |
|  | Notfallmedizin | | 12 (20,7) | 4 (33,3) | 7(17,1) | 33 (25,0) | 1 (8,3) | 1 (5,6) | 0 (0,0) | 0,347 |
|  | Schwindel | | 5 (8,6) | 2 (16,7) | 6 (14,6) | 23 (17,4) | 1 (8,3) | 6 (33,3) | 0 (0,0) | 0,282 |
|  | Funktionelle Störungen | | 3 (5,2) | 1 (8,3) | 1 (2,4) | 15 (11,4) | 2 (16,7) | 2 (11,1) | 0 (0,0) | 0,51 |
| Drittmittelanträge, n (%) | | |  |  |  |  |  |  |  |  |
|  | Erfolgreich, gesamt | | 25 (43,1) | 3 (25,0) | 8 (22,9) | 16 (12,7) | 1 (8,3) | 5 (27,8) | 1 (100,0) | **<0,001** |
|  | Noch nie gestellt | | 23 (39,7) | 8 (66,7) | 24 (68,6) | 102 (81,0) | 11 (91,7) | 12 (66,77) | 0 (0,0) | **<0,001** |
|  | Intramural, erfolgreich | | 18 (31,0) | 2 (16,7) | 3 (8,6) | 5 (4,0) | 0 (0,0) | 0 (0,0) | 0 (0,0) | **<0,001** |
|  | Intramural, nicht erfolgreich | | 11 (19,0) | 1 (8,3) | 1 (2,9) | 3 (2,4) | 0 (0,0) | 1 (5,6) | 0 (0,0) | 0,003 |
|  | Öffentlich erfolgreich | | 17 (29,3) | 1 (8,3) | 4 (11.4) | 8 (6,3) | 1 (8,3) | 3 (16,7) | 1 (100,0) | **<0,001** |
|  | Öffentlich nicht erfolgreich | | 11 (19,0) | 1 (8,3) | 3 (8,6) | 9 (7,1) | 1 (8,3) | 3 (16,7) | 0 (0,0) | 0,328 |
|  | Stiftung, erfolgreich | | 9 (15,5) | 1 (8,3) | 5 (14,3) | 11 (8,7) | 1 (8,3) | 5 (27,8) | 1 (100,0) | 0,038 |
|  | Stiftung, nicht erfolgreich | | 7 (12,1) | 0 (0,0) | 3 (8,6) | 5 (4,0) | 1 (8,3) | 1 (5,6) | 0 (0,0) | 0,476 |
|  | Industrie, erfolgreich | | 11 (19,0) | 2 (16,7) | 3 (8,6) | 8 (6,3) | 0 (0,0) | 3 (16,7) | 0 (0,0) | 0,135 |
|  | Industrie, nicht erfolgreich | | 2 (3,4) | 0 (0,0) | 0 (0,0) | 1 (0,8) | 0 (0,0) | 0 (0,0) | 0 (0,0) | 0,709 |
| Zufriedenheit mit wiss. Tätigkeit, n (%) | | |  |  |  |  |  |  |  | **<0,001** |
|  | Sehr zufrieden | | 14 (24,1) | 1 (8,3) | 2 (5,6) | 3 (2,3) | 1 (8,3) | 0 (0,0) | 0 (0,0) |  |
|  | Eher zufrieden | | 15 (25,9) | 0 (0,0) | 8 (22,2) | 15 (11,7) | 2 (16,7) | 1 (5,6) | 1 (100,0) |  |
|  | Teils teils | | 11 (19,0) | 2 (16,7) | 5 (13,9) | 20 (15,6) | 2 (16,7) | 4 (22,2) | 0 (0,0) |  |
|  | Eher unzufrieden | | 6 (10,3) | 5 (41,7) | 7 (19,4) | 29 (22,7) | 1 (8,3) | 1 (5,6) | 0 (0,0) |  |
|  | Sehr unzufrieden | | 6 (10,0) | 1 (8,3) | 0 (0,0) | 16 (12,5) | 0 (0,0) | 1 (5,6) | 0 (0,0) |  |
|  | Trifft nicht zu | | 6 (10,3) | 3 (25,0) | 14 (38,9) | 45 (35,2) | 6 (50,0) | 11 (61,1) | 0 (0,0) |  |
| Hürden bei der Wissenschaft, n (%) | | |  |  |  |  |  |  |  |  |
|  | Familie und Forschung | | 15 (25,9) | 4 (33,3) | 12 (29,3) | 37 (28,0) | 2 (16,7) | 4 (22,2) | 0 (0,0) | 0,946 |
|  | Klinik und Forschung | | 28 (48,3) | 9 (75,0) | 18 (43,9) | 70 (53,0) | 4 (33,3) | 4 (22,2) | 0 (0,0) | 0,066 |
|  | Probleme am Arbeitsplatz | | 5 (8,6) | 4 (33,3) | 8 (19,5) | 64 (48,5) | 5 (41,7) | 5 (27,8) | 0 (0,0) | **<0,001** |
|  | Fehlende Finanzierung/Vergütung | | 12 (20,7) | 6 (50,0) | 7 (17,1) | 31 (23,5) | 2 (16,7) | 4 (22,2) | 0 (0,0) | 0,362 |
|  | Fehlende Qualifikation | | 8 (13,8) | 3 (25,0) | 7 (17,1) | 28 (21,2) | 1 (8,3) | 3 (16,7) | 0 (0,0) | 0,809 |
|  | Fehlendes Thema | | 6 (10,3) | 0 (0,0) | 5 (12,2) | 10 (7,6) | 2 (16,7) | 1 (5,6) | 0 (0,0) | 0,752 |
|  | Forschungsfreundliches Umfeld | | 5 (8,6) | 4 (33,3) | 9 (22,0) | 36 (27,3) | 2 (16,7) | 3 (16,7) | 0 (0,0) | 0,125 |
|  | Fehlende MentorInnen | | 10 (17,2) | 2 (16,7) | 8 (19,5) | 38 (28,8) | 3 (25,0) | 0 (0,0) | 0 (0,0) | 0,125 |
|  | Zu viele andere Verantwortungen | | 18 (31,0) | 5 (41,7) | 13 (31,7) | 48 (36,4) | 5 (41,7) | 5 (27,8) | 0 (0,0) | 0,912 |
|  | Fehlende Perspektive | | 3 (5,2) | 1 (8,3) | 2 (4,9) | 6 (4,5) | 0 (0,0) | 2 (11,1) | 0 (0,0) | 0,881 |
|  | Fehlen des eigenen Beitrags | | 4 (6,9) | 0 (0,0) | 4 (9,8) | 10 (7,6) | 1 (8,3) | 1 (5,6) | 0 (0,0) | 0,958 |
|  | Kein Interesse | | 4 (6,9) | 1 (8,3) | 5 (12,2) | 15 (11,4) | 2 (16,7) | 5 (27,8) | 0 (0,0) | 0,382 |
|  | Keine Hürden | | 9 (15,5) | 1 (8,3) | 2 (4,9) | 2 (1,5) | 1 (8,3) | 2 (11,1) | 0 (0,0) | 0,022 |

***Anmerkungen:*** Werte sind als Anzahl und Prozent (Spaltenprozente) angegeben. **Abkürzungen:** UK L+ = Universitätsklinik mit geriatrischem Lehrstuhl; UK L− = Universitätsklinik ohne geriatrischen Lehrstuhl; PKH Peripheres Krankenhaus mit geriatrischem Lehrstuhl; PKH L− = Peripheres Krankenhaus ohne geriatrischen Lehrstuhl; RK = Rehaklinik.
